# Heat Propagation Through Fins Made of Polymeric Materials Manufactured by 3D Printing

**DOI:** 10.3390/polym18111315

**Published:** 2026-05-26

**Authors:** Florin Negoescu, Vasile Merticaru, Andrei Marius Mihalache, Vasile Ermolai, Oana Dodun, Nicolae-Răzvan Mititelu, Gheorghe Nagîț, Marius-Ionuț Rîpanu, Adelina Hrițuc, Laurențiu Slătineanu

**Affiliations:** Department of Machine Manufacturing Technology, “Gheorghe Asachi” Technical University of Iași, 700050 Iași, Romania; florin.negoescu@academic.tuiasi.ro (F.N.); vasile.merticaru@academic.tuiasi.ro (V.M.); marius-andrei.mihalache@academic.tuiasi.ro (A.M.M.); vasile.ermolai@academic.tuiasi.ro (V.E.); nicolae-razvan.mititelu@student.tuiasi.ro (N.-R.M.); gheorghe.nagit@academic.tuiasi.ro (G.N.); marius-ionut.ripanu@academic.tuiasi.ro (M.-I.R.); hrituc.adelina3295@yahoo.com (A.H.); laurentiu.slatineanu@academic.tuiasi.ro (L.S.)

**Keywords:** polymers, fins, different cross-sections, heating electrical resistance, infrared camera, distance of heating, empirical mathematical model

## Abstract

To investigate simultaneously both the effect of fin cross-sectional shape on heat transfer and the influence of different polymeric materials, test samples were manufactured by 3D printing in the form of bushings with attached radial fins of varying cross-sections. Through the research undertaken, the aim was to obtain information regarding the length of the fin at which a certain temperature is reached; therefore, the length that ensures efficient heat transfer to the external environment. Dedicated testing equipment was designed and built to test the thermal transfer in fins made of three different materials (polylactic acid (PLA)-based materials, i.e., standard PLA, PLA with carbon black (protopasta), and PLA with graphene (prografen)) and, respectively, with different sizes and shapes of the cross-section (circular, square, equilateral triangular, and rectangular). The experimental results were mathematically processed to develop empirical models that illustrate both the direction and the intensity of the influence of the input factors on the fin length at which a specific temperature is reached. Under certain conditions, radial components with a circular cross-sectional area of 20 mm^2^ showed significant differences depending on the polymer type. For the polylactic acid material, this length was 42% higher than for prografen and 25% higher than for protopasta.

## 1. Introduction

One of the groups of materials of real current interest for very different fields of activity is the polymeric materials group.

The current extensive use of polymers and composite materials incorporating polymers has led to intense study of their properties to obtain useful information for different applications. It was therefore natural for researchers to focus their attention on the heat transfer capacity of polymeric materials [1,2,3,4,5,6,7,8].

On the other hand, a technology increasingly used for the manufacture of parts from polymeric materials is 3D printing. The possibilities of modeling the internal structure of materials incorporated in parts manufactured by 3D printing have led researchers to focus their attention on the extent to which the internal structure can influence the mode of heat transfer.

Thus, Reynolds et al. proposed developing research on the influence of various factors on the heat transfer characteristics of heat exchangers manufactured by 3D printing from polymers [7]. They found that better results are obtained by using a sheet gyroid design, a certain porosity, and specific dimensions of the heat exchangers.

Ly et al. compared some thermal properties valid for two categories of heat exchangers manufactured from polymer by 3D printing [8]. They showed that the heat transfer rate per unit mass of a polymer gyroid can be higher than that of a certain grade of steel, but lower than that of aluminum.

Heat dissipation through radial fins has been investigated by various researchers in the case of finned radiator parts. Lalot et al. showed that the efficiency of a fin made of two materials can be increased by using a galvanic coating [9]. They also observed that favorable effects are obtained for large values of the ratios between the large radius of the fin tip and the radius of the fin base.

Heinle and Drummer carried out an experimental study that examined heat transfer in the case of using cooling ribs made of thermally conductive polymers [10]. They found that, under certain thermal conditions, heat transfer similar to that of metallic materials, such as copper and aluminum, is possible.

Experiments by Marchetto et al. on polymer samples have shown that there are situations in which it is possible to replace metallic materials with polymeric materials in applications involving heat transfer [11]. They highlighted the possibilities of increasing the heat transfer capacity of some polymeric materials by including reinforcements made of conductive materials.

Yu et al. developed an experimental study on heat transfer in fins with rectangular cross-sections by taking into account the length and height of the fin and the number of fins [12]. For such situations, they proposed a correlation for predicting the average Nusselt number corresponding to a radial heat sink.

The heat transfer through annular fins placed on a base part made of a different metallic material than that of the fin was investigated and modeled by Kanthimathi and Naga [13]. They found the possibility of obtaining a higher heat transfer efficiency in such situations.

An experimental investigation of the natural convection cooling process of vertical cylinders with inclined fins with rectangular section was undertaken by Lee et al. [14]. The main conclusion formulated was that the efficiency of inclined fins is 30% lower than that of radial fins.

Mathiazhagan and Jayabharathy used an analytical method to characterize the temperature distribution for fins with different cross-sectional profiles [15]. They found that the heat transfer rate is higher for fins with a triangular cross-section, followed by the pit fin and rectangular fin, respectively. A verification of the results obtained using an analytical method was carried out by comparing them with those obtained using the finite difference method.

Asadi and Khoshkho addressed the problem of temperature variation along a fin with a constant rectangular cross-section [16]. They noted that if the temperature profile is constant, an increase in the rate of heat transfer by convection and radiation becomes possible.

Khaled addressed the problem of heat transfer through different types of fins, namely fins with constant cross-sectional area, fins characterized by a constant cross-sectional area gradient, and radial fins with a power-law cross-sectional area distribution [17]. The development of theoretical models allowed him to determine that high heat transfer efficiency corresponds to fins that have a larger tip and surface areas and smaller tip thickness than those of the straight fin.

Madhura and Rajath conducted a study on the temperature variation in fins made of different materials (copper, aluminum, iron, stainless steel) and with different cross-sectional shapes (triangular, rectangular, annular, circular) [18]. They concluded that copper fins with a circular cross-section provide the highest heat transfer rate.

Heat transfer through fins with uniform, triangular, trapezoidal, and annular cross-sections was analyzed by Karwa [19], and possibilities for improving heat transfer were identified and exemplified.

Khan et al. performed a thermal transfer analysis for an inclined longitudinal porous fin with different profiles (trapezoidal, rectangular, and dovetail) using cascade neural networks [20]. They found that a dovetail profile provides the highest heat transfer rate.

Haq et al. performed and exemplified a detailed analysis of heat transfer of periodic-type variation when extended surfaces, such as fins, are used [21].

A complete numerical model describing the temperature distribution in configurations including circular, rectangular, and annular fins was proposed by Cui et al. [22]. The effectiveness of using the annular fin in terms of heat storage possibilities was highlighted. Habib et al. investigated the temperature distribution in a radial porous fin under steady-state conditions using the Levenberg–Marquard algorithm [23]. They found that the thermal model developed in this way provides good prediction accuracy.

Keshtiban et al. considered that the fin arrangement exerts a strong influence on the thermohydraulic characteristics of radiators, resorting to an analysis of the behavior of different types of radial fin arrangements [24]. The main conclusion they reached refers to the recommendation of using radial arrangements with straight fins in impact annular jet heat radiators.

Kumar et al. set out to investigate the heat transfer in a convex-shaped porous spine fin in the presence of a ternary hybrid nanofluid [25]. To obtain a mathematical solution to the problem addressed, they used the shifted Vieta–Lucas polynomials-based collocation technique. It was concluded that the porous fin located in a hybrid nanofluid ensures more intense heat transfer than a dry fin.

A study on heat transfer in heat sinks made of thermally conductive polymer-based composites was conducted by Bagatella et al. [26]. They found that boron nitride (BN) microplatelet composites fabricated by fused deposition modeling can be used in electronic devices to manage thermal transfer processes.

The Taylor wavelet method was used by Manvitha et al. to model thermal transfer by convection and radiation in a porous fin fabricated from functionally graded materials in a medium containing a hybrid nanofluid [27].

Souza et al. considered the heat transfer efficiency of heat exchangers, including a heat exchanger made of pure polydimethylsiloxane (PDMS), a heat exchanger made of a material that included recycled graphite (PDMS+Graphite 30 mass%), and a third exchanger that incorporated commercial aluminum nanoparticles (PDMS+Aluminium 30 mass%) [28]. They found that the best heat transfer was ensured in the case of the heat exchanger made of composite materials that included a PDMS matrix and recycled graphite.

Li et al. investigated the temperature distribution in polymer pin-fin microchannel heat exchangers used in electronic chipsets. They concluded that the variable-size pin-fin structures can contribute to the attenuation of the effects of so-called thermal hotspots [29].

Manvitha et al. addressed aspects regarding the thermal behavior of conical spines that perform thermal transfer to a convective-radiative and moist environment, including using the Fibonacci wavelet collocation method [30]. They found that the inclined spine exhibits higher thermal transfer efficiency compared to the straight conical spine.

It can be noted that, in principle, there have been concerns regarding how the parameters characterizing the 3D printing process of parts made of polymeric materials, such as the shape and dimensions of the parts, can influence the thermal properties of the polymeric materials used to manufacture the parts by 3D printing. Different theoretical and experimental methods have been used to investigate and model the influence exerted by the parameters of the 3D printing process of parts made of polymeric materials on heat transfer [31,32,33,34,35,36,37,38,39]. There is a direct correspondence between the parameters of the 3D printing process and some properties of the manufactured parts, such as those that define their internal structure [9,13]. Such properties will affect how heat transfers through the parts and through the components of the parts.

The consultation of the specialized literature accessible to the authors of this work has highlighted an approach mainly to aspects regarding heat transfer through fins made of metallic materials, with less information on how fins made of polymeric materials behave when they are involved in heat transfer processes [40,41,42]. Most research results mainly aim at the development of theoretical mathematical models and eventual verifications, also through other theoretical methods, with fewer concerns regarding the experimental research of heat transfer processes in the case of fins made of different materials, with different shapes and sizes. It is worth noting the use of various theoretical methods for modeling the temperature distribution along the fins, including finite element analysis [19,30], DTM-Pade Approximant [43], variational iteration method [27], computational fluid dynamics (CFD) simulations [20], Taylor wavelet method [27], the Fibonacci wavelet collocation method [30], etc.

The existence of possibilities for optimizing fin solutions to maximize the intensity of the heat transfer process or another characteristic parameter of the fins has led to research in this direction, with the development of appropriate mathematical models [12,14,17,21,23,24].

In many practical situations, a more efficient heat dissipation is achieved by increasing the size of the surfaces intended for heat transfer to the outside environment. Such an increase can also be achieved by using radial fins of different shapes and cross-sections of different sizes, these fins being connected to the body from which they receive heat. The authors of this paper were aware of only a few works that address such a problem, and the approach was made only tangentially.

On the other hand, from the point of view of industrial practice, it is of economic interest to use certain fin lengths for which efficient heat transfer is ensured. This could mean that by using too long fin lengths, an increase in material consumption could occur, without the increase in fin length contributing to a significantly faster heat transfer. For this reason, through the proposed and developed research, the aim was to obtain empirical mathematical models that would provide information on the fin length that ensures efficient heat transfer, but without unnecessarily increasing material consumption.

One problem addressed in the research whose results were presented in this article was identifying a part shape that would allow an operational illustration of the extent to which thermal transfer can be affected by the shape and dimensions of the part made of polymeric material manufactured by 3D printing. For this purpose, a part in the form of a bushing was designed with fin-type components arranged radially, in a single plane, and subjected to a heating process. The heating was provided by using an electrical resistance placed in a central part of the metallic material located inside the bushing. It was considered that recording the heat transfer with the help of an infrared camera would make it possible to directly highlight the extent to which the shape and dimensions of the cross-section through the radial component could provide additional information regarding the heat transfer through the fin. The modeling this process using the finite element method proved the validity of the previously formulated hypothesis. The design of the equipment that allows the experimental verification of the aforementioned hypothesis also facilitated the determination of empirical mathematical models. These models illustrate the direction and intensity of the influence exerted by the nature of the material, as well as the shape and dimensions of the cross-sections through the radial components, on how heat propagation through the radial fins takes place.

## 2. Materials and Methods

### 2.1. Initial Considerations

The proposed experimental research aimed to obtain additional information on the propagation of heat through solid bodies in the form of fins with different shapes and dimensions of their cross-sections, which are subjected to heating at one of their ends.

**Figure 1:**
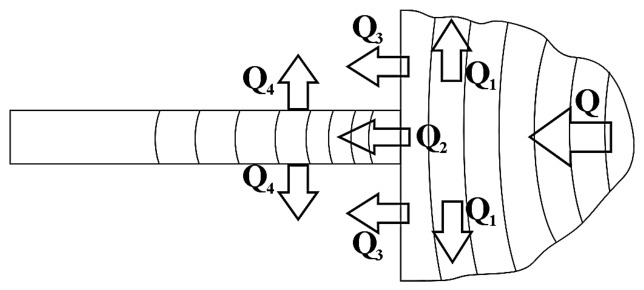
Heat transfer in the case of a fin-shaped component.

A graphic representation of the technical solution from which it was started can be seen in Figure 1. A component of a basic solid body in the form of a fin was considered that receives a heat flux from the end through which the fin is embedded in the solid body.

Assuming that this base body is heated internally by a heat source, in the area near the fin, there will be heat transfer by conduction from the base body to the fin-shaped component and by convection to the surrounding environment, provided that the temperature at the base of the fin is not too high. A heat balance equation corresponding to such a situation will have the form:

*Q* = *Q*_1_ + *Q*_2_ + *Q*_3_ + *Q*_4_ (1)

where *Q* is the amount of heat that propagates by conduction through the base body to the fin-shaped component;

*Q*_1_—the amount of heat transmitted by conduction to the peripheral area of the base body;

*Q*_2_—the amount of heat transmitted by conduction through the fin;

*Q*_3_—the amount of heat transmitted, especially by convection, to the environment by the peripheral area of the base body;

*Q*_4_—the amount of heat transmitted, especially by convection, to the environment by the fin.

If at the beginning of the heating process, the amounts of heat *Q*, *Q*_1_, *Q*_2_, *Q*_3_, and *Q*_4_ increase, after a certain time, a situation of stationary heat flow will be reached, characterized by constant values of the mentioned amounts of heat.

It is assumed that the heat quantities *Q*_3_ and *Q*_4_ are relatively small. Such a situation would correspond to the beginning of heat propagation. This would mean that, along the fin-shaped component, there would be a temperature decrease from the area of incorporation in the base body towards the free end of the fin. The temperature variation could be observed with the help of an infrared camera.

**Figure 2:**
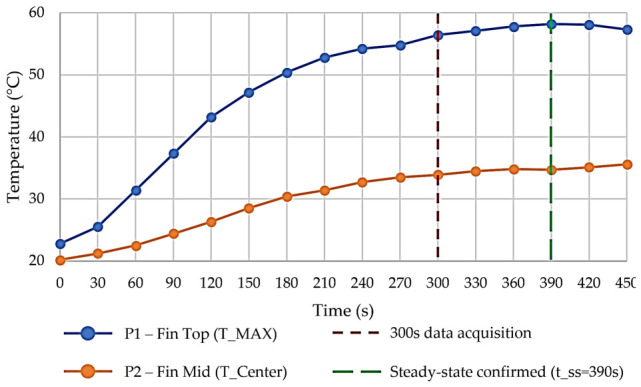
Temperature–time curves at fin top (P1, *T_max_*) and fin mid-length (P2, *T_center_*) (standard PLA, circular cross-section—28.274 mm^2^; red dashed line—5 min acquisition point (300 s) used in the manuscript; green dashed line—experimentally confirmed steady-state, *t_ss_* = 390 s, Δ*T* ≤ 0.5 °C, emissivity *ε* = 0.85).

As will be highlighted later, in the experimental study of the temperature variation along the radial fin, it was considered that after about 6 min from the start of the heating process, a stationary thermal regime is reached (Figure 2). Adopting this simplifying hypothesis, it was no longer necessary to take into account time as an independent variable in tracking the temperature distribution mode with the help of the infrared camera.

To verify that quasi-steady-state thermal conditions were established before image acquisition, a dedicated experiment was performed, using the same equipment and the same geometry of one of the samples (standard PLA, circular cross-section, *A* = 28.274 mm^2^). The temperature was monitored at two reference points—at the top of the fin (P1) and at the middle of the fin (P2)—at 30 s intervals from start-up, up to 450 s. Since the heating element was regulated by an on/off controller, the system did not approach a true steady state, but a periodic thermal quasi-steady state. Consequently, the steady state was characterized as the condition in which the average temperature of the cycle at both reference points varied by no more than 0.5 °C between successive 30 s readings. This corresponds to a threshold five times higher than the thermal noise threshold of the chamber (≤0.1 °C) and well below its absolute accuracy (±2 °C). The global capacity model (Bi = V h/(A_s k) = 0.077 < 0.1, according to [32]) provides an upper-bound theoretical time constant of *τ* ≈ 270 s for constant boundary conditions. Fitting the experimental curve to the transient data yields apparent time constants of τ_P1 ≈ 146 s and τ_P2 ≈ 227 s, consistent with the expected acceleration due to the active controller. Infrared images for *l_θ_* determination were acquired at *t* = 300 s (5 min), at which time the fin temperature at P1 had risen to approximately 85% of the adjusted quasi-steady state. The combined uncertainty of the *l_θ_* length measurement, derived from the thermal accuracy of the camera (±2 °C), the local temperature gradient along the fin, and the spatial resolution (IFOV = 3.75 mrad at *d* ≈ 300 mm), was estimated to be ±1.26 mm, and is therefore negligible compared to the reported fin lengths (20–60 mm).

Another simplifying hypothesis in determining empirical mathematical models regarding the temperature distribution in radial fins made of polymeric materials manufactured by 3D printing was that of not taking into account the heat transfer by radiation from the radial fins to the external environment. In such situations, thermal radiation is dependent on the thermal emissivity characteristics of the fin material. Such simplifying assumptions have also been adopted by other researchers interested in the problem of temperature distribution in radial fins [19,25,38,44,45,46].

**Figure 3:**
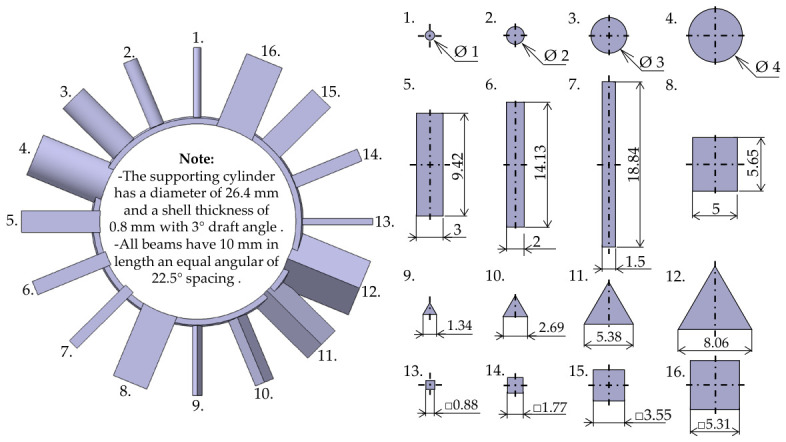
Top view of the test sample showing radial components with different cross-sections.

Taking into account the previous hypotheses, a test sample was designed in the form of a solid base body provided with several fin-shaped components arranged along radial directions (Figure 3). The radial fin-shaped components will have cross-sections with different shapes and sizes, with circular, square, equilateral triangle, and rectangular cross-sections being used. Under such conditions, it will become possible to observe the way heat propagates through the radial component with distinct shapes and areas of cross-sections. An advantage of such a solution is that it allows for approximately the same heating (same temperature) in the areas where the fin-shaped components are embedded in the base body.

It is assumed that the rate of heat propagation through the radial component will be influenced by several factors and groups of factors, such as:

- Shape and dimensions of the cross-section through the fin-shaped component;
- Thermal conductivity of the material from which the base body and the fin-shaped component are made, as well as other thermal characteristics of this material (such as, for example, specific heat);
- The amount of heat transmitted from the base body to the radial component and the speed at which this transmission occurs;
- How the process of heat transmission from the fin-shaped component to the external environment takes place. The presence of cold air currents in contact with the outer surface of the radial fin will change the way heat propagates through the radial fin-shaped component. It is expected that the heat from the component will be taken up more easily and faster by the air currents by radiation, and, as such, an increase in the speed of heat propagation through the radial fin will also occur;
- The internal structure of the material of the radial fin-shaped component. For example, the existence of gaps of different sizes will affect the rate of heat transfer through the radial fin component;
- The existence, near the fin-shaped component, of heat sources whose radiation could reach the fin-shaped component, etc.

The widely known form of Fourier’s law for a radial fin describing the heat flux *Q* is [46]:

(2) $$Q=-kA\frac{dT}{dx}$$

where *k* is the thermal conductivity of the fin material, *A* is the cross-sectional area of the fin, and *dT*/*dx* is the temperature gradient along the fin. A similar equation allows us to see that the larger the cross-sectional area, the smaller the length *l* of the distance at which the temperature is evaluated, and the higher the thermal conductivity of the fin material, the more intense the heat transfer.

Heat transfer according to the situation under consideration has been previously studied and modeled for the case of fins used for heat dissipation in various categories of radiators, ventilated rotors, electronic components, etc. Some mathematical models that can be used for the design of fins are part of the classical theory of heat transfer. They have even been the subject of some standards or other categories of regulations.

Thus, the general differential equation corresponding to the heat transfer in the case of a fin with a variable cross-section can be derived by taking into account Fourier’s law and the energy balance. Such an equation can have the form [47,48]:

(3) $$\frac{d}{dx}\left(kA\left(x\right)\frac{dT\left(x\right)}{dx}\right)-hP\left(x\right)\left[T\left(x\right)-T_{\propto }\right]-\varepsilon \sigma p\left(x\right)\left[T^{4}\left(x\right)-T_{\infty }^{4}\right]=0$$

where *A*(*x*) is the cross-sectional area of the fin, *x*—the distance from the base of the fin, *p*(*x*)—the perimeter of the cross-sectional area of the fin, *h*—the convection heat transfer coefficient at the fin surface, *ε*—the emissivity, *σ*—the Stefan–Boltzmann constant, and *T_∞_*—the temperature of the surrounding medium.

The solution to such an equation is:

(4) $$T\left(x\right)=C_{1}e^{mx}+C_{2}e^{-mx}$$

where *C*_1_ and *C*_2_ are constants, and *m* is a characteristic parameter of the fin. The value of *m* can be determined using the equation:

(5) $$m=\sqrt{\frac{hP}{kA}}$$

where *h* is the convective heat transfer coefficient, *P*—the perimeter of the fin cross-section, *k*—the thermal conductivity of the fin material, and *A*—the fin cross-section area.

Variants of Equation (3) have also been mentioned, but sometimes without taking into account the last term on the left-hand side of the equation [49,50,51,52].

On the other hand, an even simpler variant of the equation is known that highlights the temperature variation along the fin. This equation considers a fin exposed to a flowing fluid, capable of determining the temperature variation *T*(*x*) through a fin with a constant cross-sectional area, along the *x*-axis, from the wall to which the fin is attached [53,54,55,56]:

(6) $$\frac{d^{2}T\left(x\right)}{dx^{2}}-m^{2}\left(T\left(x\right)-T_{\infty }\right)=0$$

where *T_∞_* is the fluid temperature, and for *m*^2^, the relationship is valid; Equation (7) is valid.

(7) $$m^{2}=\frac{hP}{kA}$$

In the case of radial fins with circular cross-sections of radius *r*, Equation (1) becomes [45]:

(8) $$\frac{1}{r}\left(r\frac{dT}{dr}\right)-m^{2}\left(T-T_{\infty }\right)=0$$

Assuming that there is a non-Fourier heat conduction in the convective–radiative fin and by adopting mixed boundary conditions, Ma et al. [35] proposed the use of an equation of the following form to highlight the temperature variation along the fin:

(9) $$T\left(x\right)=\frac{\cos h\left(N_{c}x-N_{c}\right)}{\cos h\left(N_{c}\right)}$$

where *N_c_* is the coefficient of fin, *h* being a convective parameter.

Close in form to Equation (9) is the equation proposed to be used when the length *L* of a straight fin with an adiabatic tip is known [32,33,39]:

(10) $$T\left(x\right)=T_{\infty }+\theta_{b}\frac{\cosh\;\left[m\left(L-x\right)\right]}{\cosh\;\left(mL\right)}$$

If the temperature value *T*(*x*) is known, a mathematical relationship can be deduced from Equation (10) that highlights the distance *x* from the base of the radial fin at which a certain temperature *T*(*x*) is reached, considering that it is a straight fin with an adiabatic tip:

(11) $$x=L-arc\;cosh\left\{\left[T\left(x\right)-T_{\infty }\right]\left[\cosh\left(mL\right)\right]/\theta_{b}\right\}$$

For a straight fin, in the case of convection heat transfer and when the fin tip is not adiabatic, the temperature variation corresponds to a relationship of the form [13,32,33,39]:

(12) $$T\left(x\right)=T_{\infty }+\theta_{b}\frac{\cos h\left[m\left(L-x\right)\right]+\frac{h}{mk}\sin h\left[m\left(L-x\right)\right]}{\cos h\left(mL\right)+\frac{h}{mk}\sin h\left(mL\right)}$$

where *L* is the length of the fin.

It is known that, at present, some parts of polymeric materials can be made by additive manufacturing processes, characterized, for example, by the successive addition, in different ways, of layers of molten material. Given the similarities of such processes with those of classical flat printing processes, the use of the concept of 3D printing processes has become widespread. One such process in the previously mentioned category is called fused deposition modeling (FDM), which allows obtaining a very wide range of products made of polymeric materials with different shapes and sizes. The mentioned process (FDM) has the property of facilitating the creating of an internal structure of the material of the printed part, with variations between certain limits. This is possible by modifying the values of the parameters that characterize the 3D printing process [9,13]. Currently, it is considered that the number of input factors in a 3D printing process is very large, with just a few of these factors being the following:

- The temperature of the nozzle through which the material, in the form of a wire, is extruded (for example, 210 °C);
- The diameter of the cylindrical hole in the nozzle, respectively, the extrusion width (for nozzles with holes that have other cross-sectional shapes);
- Printing speed, with distinct values for different areas of the part being generated;
- The degree of filling/infill;
- The temperature of the table on which the layers of molten material are laid down;
- The thickness of the layer of molten material, etc.

The transmission of heat from the end of the component, through which it is embedded in the solid base body, to the free end of the component should determine a gradual decrease in temperature towards the free end of the embedded component (Figure 1). This decrease in temperature could be observed and highlighted with an infrared camera, the objective of which should be directed towards several components embedded in the solid base body. Using cross-sections with different profiles and component sizes, the images taken with the help of the infrared camera should provide information regarding the amounts of heat transmitted through components with cross-sections having different profiles and areas.

**Figure 4:**
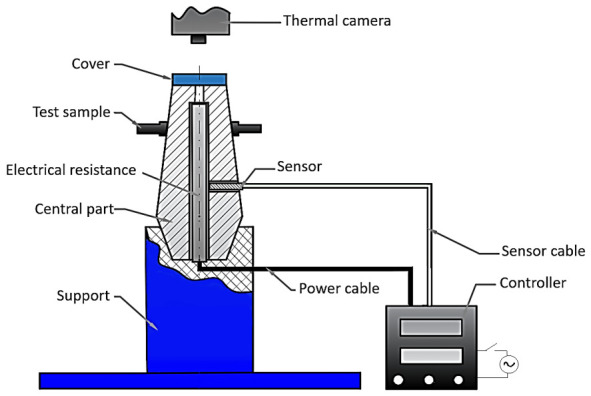
Schematic representation of the equipment originally designed to highlight heat transfer through radial components with different shapes and cross-sectional areas.

To carry out the experimental research, the use of adequate equipment was considered. The schematic representation of this equipment can be seen in Figure 4. The equipment involves the use of a polylactic acid support part. A central steel part was immobilized in the support part, provided with a bore in which an electrical resistance is inserted. The electrical resistance will contribute to the heating of the central part. From the central part, the heat will be transmitted to the test sample provided with radial fins. The electrical resistance is powered from the usual alternating current network using a controller. A sensor contributes to the measurement of the temperature. A controller will act in such a way as to maintain, as much as possible, a preset temperature value. The assumption was made that there is a uniform heat transfer from the electric resistance to the core. This uniformity of heating of the core was highlighted, to a certain extent, by the images taken by the thermal camera. In reality, after reaching the maximum preset temperature value, the controller stops the power supply to the electric resistance; the temperature drops to a minimum preset value, after which the electric resistance is reconnected to the power supply circuit.

To reduce the propagation of heat to the external environment, a polylactic acid cover was used. This also contributes to an improvement in the quality of the images taken with the help of the infrared camera.

### 2.2. Modeling by the Finite Element Method of Heat Propagation Through Bodies with Different Cross-Sections

The aim was to design a virtual setup model that would replicate results obtained by means of experimental testing. When assessing thermal stress problems, one must account for the fact that temperature distribution inside thermal analyses may induce thermal strains in a structural analysis. If the coupling is aimed for, the temperature values resulting from a thermal analysis are mapped to a structural one as a thermal load. Problems are found in the case of dissimilar meshes, making it desirable to use a mapping technique that transfers temperatures to the structural model. When using coupled-field type of analyses, there is no need for separate analyses with transferred loads because they allow for fully coupled thermo-mechanical problems. The 3D geometry was saved in Parasolid format for translation to finite element software. Ansys 2024 R1 was used, and the researcher licensed the coupled-field static module for the application of the finite element method (FEM). This method considers both displacements and temperatures as degrees of freedom inside the same element and is best suited for cases with internal heat generation caused by mechanical deformation.

The material was retrieved from the software library. The major setback of this approach is a lack of engineering data in the form of datasheets obtained using experimental tests regarding thermal-dependent stress–strain evolution for polymers used in fused deposition modeling.

To overcome this, experimental results were used. From previous work, data from tensile testing of FDM-printed PLA were retrieved. Samples were printed flat, on the side, and in vertical positions. Tests were performed on an Instron 4411 uniaxial testing machine with a load capacity of 5 kN. For the current research, data resulting from horizontal and vertical testing were used. To address the anisotropy induced by the fused deposition modeling process, two distinct material definitions were created—PLA FDM Horizontal and PLA_FDM_Vertical—corresponding to the two principal printing orientations used. The input data for each material were derived from uniaxial tensile tests performed on specimens printed along the horizontal (*XY*) and vertical (*Z*) directions. Within ANSYS Engineering Data, the material was defined as orthotropic elastic, enabling the assignment of direction-dependent stiffness parameters. For the horizontally printed PLA, the in-plane elastic moduli (*E*_1_ and *E*_2_) were set higher than the out-of-plane modulus (*E*_3_), reflecting the stronger inter-filament bonding within the printed layers compared to the inter-layer adhesion. Conversely, for vertically printed PLA, the through-thickness modulus (*E*_3_) was reduced to capture the weaker layer adhesion and increased susceptibility to delamination under tensile load. The local coordinate system for each material domain was oriented according to the filament deposition direction, ensuring that the numerical model correctly aligned the anisotropic axes with the physical printing directions. This approach allows the simulation to distinguish between the mechanical response parallel and perpendicular to the printing layers, thus integrating the print-induced anisotropy directly into the finite element formulation.

Furthermore, the coupled-field configuration enabled the inclusion of temperature-dependent effects. From previous work, results from thermogravimetric analysis, a procedure in which the material is heated until it degrades, were used to calculate mass losses. The analyses were carried out on a Thermal Analysis System TGA 2 from Mettler Toledo, Greifensee, Switzerland. In the present study, thermo-gravimetric analyses (TGA) were performed to characterize the temperature-dependent behavior of the PLA material. The experimentally observed mass loss was correlated with a reduction in material density, which directly affects thermal expansion, heat capacity, and conductivity. While TGA alone cannot measure specific heat or thermal expansion, its data are essential in identifying the temperature range where significant material changes occur. When integrated with differential scanning calorimetry/thermomechanical analysis (DSC/TMA), results provide a validated basis for reliable property modeling in finite element simulations. This link can be incorporated into the FEM model by defining temperature-dependent material properties (*ρ*(*T*), *α*(*T*), *k*(*T*)) derived or scaled from TGA data. Out of the three, only the coefficient of thermal expansion *α*(*T*) has been used, which can be scaled inversely with the residual mass fraction, since microstructural degradation leads to volumetric instability. This formulation ensures that the FEM material model reflects experimentally validated, temperature-dependent variations of PLA properties up to the onset of degradation, while providing a consistent representation of the structural and thermal deterioration observed during mass loss.

Upon import, the 3D model was assigned the modified material with an initial ambient temperature of 21 °C.

Mesh-wise, a patch-conforming method was applied with an imposed element order as quadratic. This results in only the Tet10 (10-node tetrahedral) type of elements. Advantages include more accurate stress and strain predictions, a better representation of curved geometry, and reduced mesh dependency and numerical stiffness. The ten nodes are distributed as follows: four at the corners and six at the midpoints of the edges.

A mesh sensitivity study was conducted for all three material configurations examined in the present work across six refinement levels per configuration, with global element sizes ranging from 5.0 mm to 1.0 mm. This was achieved by means of an ADPL script, which produced the number of nodes, number of elements, maximum equivalent von Mises stress, and total deformation. The maximum total deformation was found to be fully mesh-independent throughout the entire refinement range for all configurations, with variations not exceeding 1.11% across all discretization levels and values stabilizing between 0.0089 mm and 0.0091 mm, confirming that the displacement field is insensitive to mesh density within the explored range. For the maximum equivalent von-Mises stress, convergence satisfying the adopted 5% criterion was demonstrated individually for each configuration. For the PLA FDM horizontal orientation, a well-defined convergence plateau was identified spanning the 3.0 mm to 2.0 mm range, with three consecutive stress changes of 4.91%, 3.66%, and 4.78%, all satisfying the criterion and forming a tightly clustered, internally consistent band; the 2.5 mm mesh (286,548 nodes, 196,382 elements) was selected. For the PLA FDM vertical orientation, a clean convergence window was identified between 3.0 mm and 2.0 mm, with consecutive stress changes of 4.19% and 3.84% forming a monotonically decelerating sequence; the two preceding transitions at 5.40% and 5.49% exceeded the threshold by less than half a percentage point each and exhibited near the same values, indicating a stable stress field approaching convergence in a controlled and consistent manner from above; the 2.5 mm mesh (292,248 nodes, 202,919 elements) was accordingly selected. Across configurations, the converged mesh corresponds uniformly to the 2.5 mm element size, lending strong internal consistency to the mesh selection. Results at the 1.0 mm refinement level exhibited anomalous stress increases of 16.28% and 20.72%, respectively, while deformation values remained entirely stable, indicating numerical amplification of localized stress concentrations at geometrically complex hub-fin interface regions rather than genuine physical stress field changes, consistent with the elevated element skewness values documented in the mesh quality assessment. The complete mesh sensitivity results are summarized in Table 1.

The analysis settings considered a single time step with a large deflection. Non-linear controls were left program-controlled. Solver tolerance was set to 0.0000001 W/mm^2^ since, in the case of extreme heating, heat flux in finite element analysis is considered to range from 0.001 to 0.1 W/mm^2^. Temperatures were added as boundary conditions. The approximate values obtained for PLA samples were used for the inner surface of the cylindrical cone, and the default ambient temperature value was applied to all ends. In the finite element model, the thermal loading was represented not through simulation of the heat generation and conduction pathway, which would require explicit characterization of the steel–PLA thermal interface, including any intermediate grease layer, but rather through the direct prescription of experimentally measured temperature values as Dirichlet boundary conditions on the inner surface of the PLA cylindrical bore. The authors acknowledge that the spatial uniformity of the temperature boundary condition represents a simplification relative to the physical apparatus, in which the cyclic on-off control of the electrical resistance and the geometric asymmetry of the bore may introduce non-uniformity in the surface temperature distribution.

**Figure 5:**
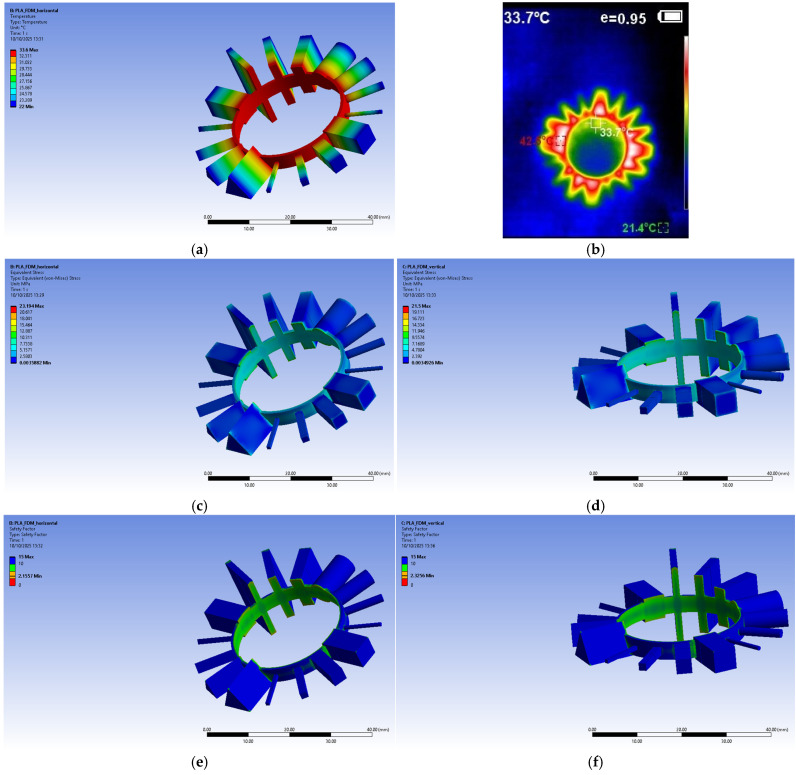
Plots of results: (**a**) temperature distribution for PLA FDM Horizontal; (**b**) image captured by the thermal camera during experimental tests; (**c**) equivalent von Mises stress distribution for PLA FDM Horizontal; (**d**) equivalent von Mises stress distribution for PLA_FDM_Vertical; (**e**) safety factor for PLA FDM Horizontal; (**f**) safety factor for PLA_FDM_Vertical.

Results were requested in the form of temperature and other data. As can be seen in Figure 5a, the temperature distribution matches, in terms of visual assessment, the one captured by the thermal camera (Figure 5b) at an approximate value of 33 °C. In the PLA FDM Horizontal scenario, equivalent stress evaluated under von Mises criteria peaks at 20.949 MPa (Figure 5c), while for the PLA_FDM_Vertical, it registers 20.04 MPa (Figure 5d). Thermal error appears at each arm joint with the inner ring. In the PLA FDM Horizontal scenario, it registers 0.00055036, and for the PLA FDM Vertical scenario, we get 0.00051128. Thus, the safety factor, which evaluates the combined effects of thermal and mechanical stresses, registers at 2.1557 for PLA FDM Horizontal (Figure 5e) and 2.3256 for PLA_FDM_Vertical (Figure 5f).

The equivalent stress tool may show high local stress with a sharp gradient, a reliable indicator of stress risers, which can lead to crack initiation or fatigue failure, typically found in cutouts or holes. When evaluating load paths, von Mises stress distributions show how forces flow through the structure. Design implication-wise, the material could be removed from low-stress regions or added where needed. This is one of the reasons FEM was used for this study.

The classical fin equations provide the theoretical basis for identifying the governing physical parameters and for establishing the expected qualitative character of temperature decrease along the radial fin from base to free end. The FEM simulation serves as the numerical implementation of the same governing equations applied to the actual geometry, material anisotropy, and boundary conditions of the 3D-printed PLA samples. The qualitative agreement between the FEM temperature distribution and the infrared thermography results is seen by the authors as verification that the experimentally observed thermal field is physically coherent with the behavior predicted by classical fin theory, thereby providing confidence in the experimental data and their subsequent empirical modeling.

Shanmugam et al. [4] showed that the thermophysical properties of FDM-printed polymeric materials exhibit variations as a function of temperature. Changes occur in the vicinity of the glass transition temperature (*Tg*) of the base polymer, where alterations in molecular chain mobility and phonon scattering mechanisms substantially modify heat transport behavior. For PLA, *Tg* is typically reported in the range of 55–65 °C, and the experimental conditions of the present study impose that the maximum preset temperature of the central steel part is limited to 60 °C. This means that the fin specimens were, during the course of the measurements, traversing a temperature range extending from approximately 21 °C at the free end to values approaching or intersecting the lower boundary of the *Tg* range at the fin base. The authors acknowledge the limitation and emphasize that it is physically plausible only at the lower end of the temperature range, where molecular chain configurations and void fraction are relatively stable; it becomes progressively less defensible as the fin base temperature approaches 46.3 °C, the highest threshold temperature recorded in the experimental dataset.

The results of Blanco et al. [37] demonstrated that, even within the sub-*Tg* operating regime, both specific heat capacity and thermal conductivity of printed PLA parts exhibit measurable temperature dependence, driven by local crystallization phenomena, changes in void content, and the temperature sensitivity of filament-to-filament contact resistance. The addition of thermally conductive fillers, such as carbon-based materials in the case of both protopasta and prografen, was shown to alter not only the magnitude but also the temperature sensitivity of the effective thermal conductivity, meaning that the assumption of a single constant *k* is expected to introduce larger errors for the filled materials than for unfilled PLA.

The inter-layer contact resistance, arising from the incomplete wetting and partial polymer chain diffusion across the interface between successively deposited layers, constitutes a significant additional thermal resistance in the build direction that has no equivalent in the raster direction, as found by Prajapati et al. [57], a scenario that FEM simulation does not account for. The implication for the present study is direct because the radial fin components are printed by the FDM process; heat flowing from the fin base to the fin tip does so along a path that intersects both raster-direction and build-direction thermal pathways, and the effective thermal conductivity experienced by the heat flux will be an anisotropic weighted average of the two directional components.

As shown by Ravoori et al. [58], the effective build-direction thermal conductivity depends strongly on the infill percentage of the printed part. Samples printed at maximum infill exhibit higher effective thermal conductivities but still retain a measurable and significant thermal anisotropy due to residual inter-layer contact resistance that cannot be eliminated by infill maximization alone. The study also reveals that the effective thermal conductivity of FDM-printed specimens varies measurably between samples produced under nominally identical printing conditions, owing to variability in void nucleation, filament-to-filament contact quality, and the thermal history experienced by each deposited layer. The current FEM model is based on experimentally retrieved mean values for some material and boundary condition parameters, and as such, the predicted temperature distribution may diverge from the observation on any single printed specimen due to sample-to-sample property variability inherent to the FDM process.

### 2.3. Conditions for Conducting Experimental Tests

The aforementioned hypotheses were taken into account in the design of the equipment that would allow for the fulfillment of the aforementioned conditions (Figure 4). It can thus be noted that the use of polymer material test samples with a shape appropriate for the objective pursued by the experimental research is essential.

The main dimensions of the test samples made from three different polymer materials are shown in Figure 3.

The samples were manufactured by 3D printing using the fused filament fabrication process on a Bambu Lab A1 printer (Shenzhen, China). The values of the main input factors in the printing process were as follows: nozzle orifice diameter: 0.4 mm; printing speed of the first layer: 50 mm/s; printing speed of the area corresponding to the outer perimeter: 100 mm/s; printing speed of the areas corresponding to the inner perimeters: 150 mm/s; printing speed of the support structures: 150 mm/s; printer bed temperature: 60 °C; initial layer thickness: 0.2 mm, to prevent possible inconveniences related to the adhesion of the molten material to the printer bed; thickness of the other layers of the sample: 0.08 mm. The resulting part process parametrization was done using Orca Slicer V2.3.2 release candidate (open-source project, created by GitHub user “SoftFever”).

**Figure 6:**
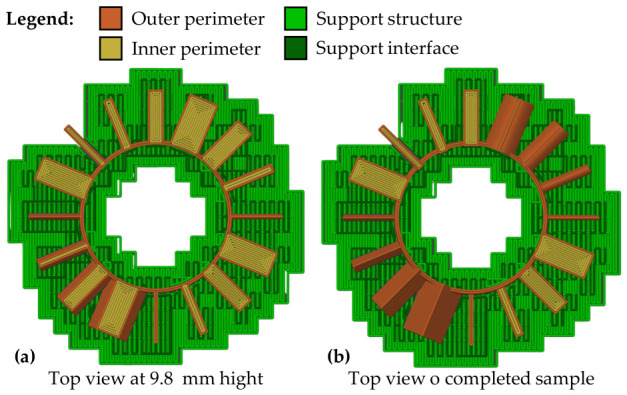
The part’s printing structure preview: (**a**) top view of the part at a height of 9.8 mm; (**b**) top view of the completed part.

It was decided to increase the number of areas with certain perimeters so that the resulting part would have maximum density. Such an approach was necessitated by the existence of cross-sections of the radial fins with different shapes and sizes. An image of the internal structure corresponding to the 3D printed samples can be seen in Figure 6.

**Figure 7:**
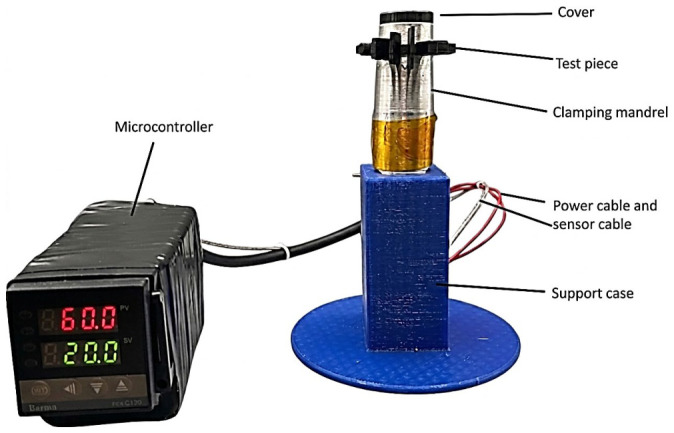
Image highlighting the placement of the test sample in the central part of the equipment.

**Figure 8:**
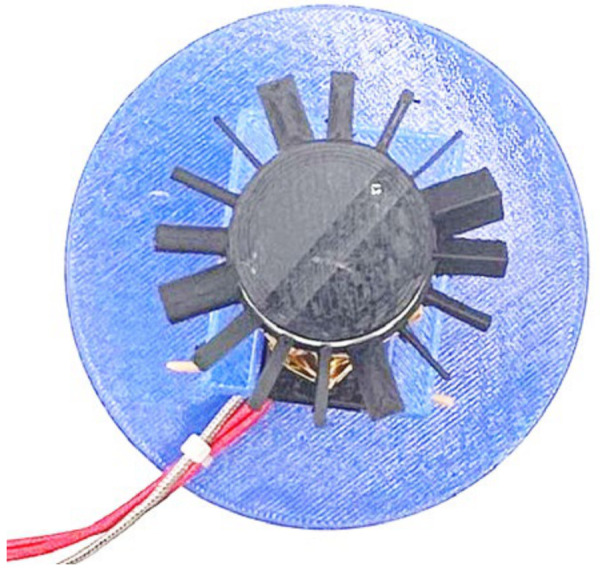
The top image highlights the placement of the test sample in the central part.

**Figure 9:**
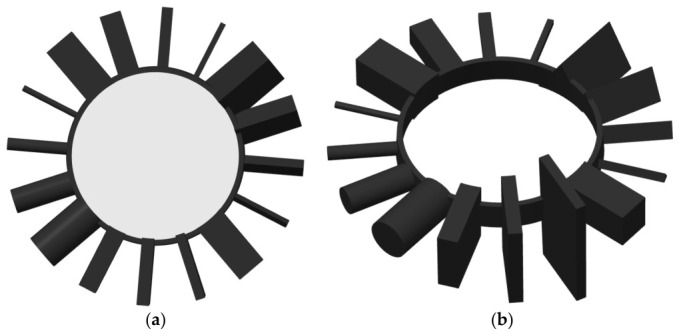
Top view (**a**) and isometric view (**b**) of the test sample.

Through the surface corresponding to its conical bore, each of the printed test samples came into contact with a central steel part (Figure 7 and Figure 8). A conical bore was used, taking into account the hypothesis that such a bore ensures a larger contact surface between the steel central part and the solid base body of the polymer material test sample (Figure 8 and Figure 9). There is practically no free space between the central part and the test sample. To further facilitate the transfer of heat from the central part to the base body of the polymer material test sample, thermally conductive grease was introduced between the two parts. Inside the central steel part, an electrical resistor powered by an alternating current source (220 V) via a temperature controller was placed (Figure 7). The controller allows us to adjust the heating temperature value of the electric resistance and the central steel part by using a temperature sensor located in the central steel part.

The central steel part of the equipment has, in the upper area, a bore into which a screw can be inserted to allow the electrical resistance to be pushed outwards when, due to different working conditions, it could get stuck inside the central part.

An HTI HT-18 thermal imaging camera, produced by Dongguan Xintai Instrument Co., Ltd., Dongguan, China, was placed above the central part. The basic body of the camera was made of polymeric material, with the images obtained using it providing information on heat transmission through fins that have cross-sections with different profiles and areas. It has a vanadium oxide uncooled infrared focal plane detector with a resolution of 256 × 192 and a cell size of 12 μm. Measurement accuracy varies from −15°C to 550°C with a ±2% error and a temperature measurement resolution of 0.1°C. Calibration involved adjusting the emissivity value to match the object being measured; thus, for matte surfaces, a default of 0.95 was used.

As an output parameter of the investigated process, the size of the distance *l_θ_* at which a certain temperature *θ* was reached was taken into account. The values of this distance were determined by punctually examining the images taken with the thermal video camera. It was noted that the values of the distance *l_θ_* will depend on the nature *m* of the test sample material, the shape and dimensions of the cross-section of the radial component, and the time interval elapsed from the moment of the start of heating the electrical resistance (from the connection of the electrical resistance to the alternating current source circuit). The distances at which temperatures of 46.3 °C (*l*_46.3_), 40 °C (*l*_40_), 33 °C (*l*_33_), and 28 °C (*l*_28_) were reached were measured.

The results of preliminary experimental tests showed that when certain values of temperature in radial fins are exceeded, softening and even plastic deformation of the components occur under the action of the fin material’s own weight. For this reason, a limitation of the temperature set using the thermal controller to values of 60 °C was resorted to.

As materials for the test samples in the form of bushings with radial components, polylactic acid (PLA) (Easy PLA, produced by the Polish company Fiberlogy, Brzezie 387, 32-014, Poland), PLA protopasta (manufactured by the American company Protopasta), and polylactic acid (PLA) reinforced with graphene (manufactured by the Polish company Prografen) were selected. The selection of materials for the test samples was carried out based on the assumption that their known thermal properties are quite different. This would allow for better separation of the experimental results to be obtained. The values corresponding to some physical-mechanical properties of these materials are those presented in Table 2. The values of tensile strength, maximum elongation, and Young’s modulus, as well as the features of use, were those mentioned by the material manufacturers. The density of the material in the samples was determined by measuring the masses and volumes of the samples. The ridges have a length of 10 mm with an angular spacing of 22.5° between them. The radial fins were placed on a center bush with an internal diameter of 26.4 mm and a height of 10 mm, with a draft angle of 3° and a shell thickness of 0.8 mm (see Figure 3).

An experimental design was used so that fins made of three different polymer materials with different cross-sections and cross-sectional areas could be tested. Information on the values of these input factors has been included in Table 3. The table shows both the dimensions of the cross-sections through the radial fins and the ratio *r* between the perimeter *p* and the area *A* of the cross-section for each type of cross-section. It was considered that this ratio, *r,* could provide useful information for highlighting the influence exerted by the dimensions of the cross-section on the way in which heat transfer occurs through the fin.

## 3. Results

**Figure 10:**
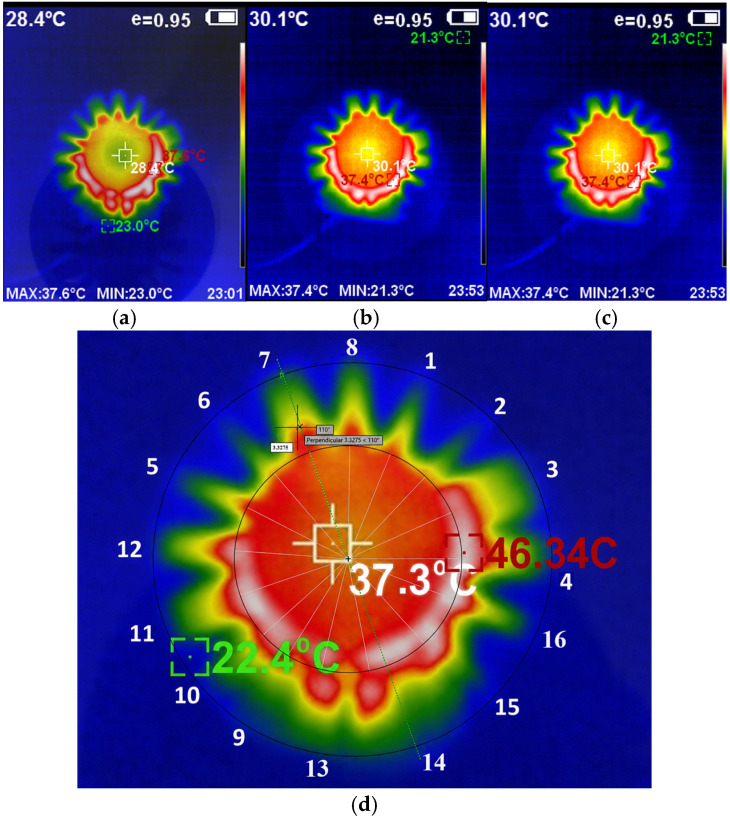
Examples of images obtained with the help of the infrared camera: (**a**) PLA sample; (**b**) protopasta sample; (**c**) prografen sample; (**d**) length measurement module *l_θ_* using AutoCAD software.

The values of the output parameter of the investigated process (the size of the distance *l_θ_* at which a certain temperature *θ* was reached, concerning the outer diameter of the bushing corresponding to the base body of the test sample) were entered in columns 7–10 of Table 3. The distances *l_θ_* were measured by taking into account the images obtained with the help of the infrared camera and using the Autodesk AutoCAD 2026 Education design software. In Figure 10, some images obtained using the infrared camera can be observed. In columns 7–10 of Table 3, the distances *l_θ_* at which temperature values of 43.6 °C, 40 °C, 33 °C, and 28 °C are recorded were entered, in principle. According to the numerical information in Table 3, the temperature of 43.6 °C was generally reached only in the case of radial fins with large areas of their cross-sections. When the value 0 (zero) was entered in the table, this means that the respective temperature was reached at a distance *l_θ_* of 0 mm. In the column corresponding to a certain temperature *θ*, no temperature value was entered when the temperature corresponding to the column was not reached. It was also observed that in the case of radial fins with large cross-sectional areas arranged in positions close to each other, a more pronounced heating of the radial fins’ embedding areas in the base bodies is noticeable.

The aim was to determine empirical mathematical models that would highlight the direction and the intensity of the influence exerted by the nature *m* of the material of the test sample, the shape *f* of the cross-section through the radial pin and the temperature *θ* on the length *l_θ_* where the temperature *θ* is reached. MATLAB 2025b software was used to process the experimental results. In the empirical mathematical models determined, heat losses to the exterior by radiation were not taken into account, as these were considered relatively low in magnitude in relation to heat transfer by conduction to the radial fins.

Under the aforementioned conditions, empirical mathematical models of power function type were proposed to predict the variation of length *l_θ_* depending on the input factors taken into account in the experimental tests:

- For the polylactic acid test sample:

(13) $$\;l_{\theta }=4198052.56f^{0.377}r^{-0.588}\theta^{-4.178}$$

In the case of the polylactic acid material, for the empirical mathematical model determined, the residual sum of squares had the value *RSS* = 58.32, the normal Gauss sum *S_G_* = 527.35, and the coefficient of determination *R*^2^ = 0.773. According to currently accepted considerations, for a value of the *R*^2^ determination coefficient between 0.7 and 0.9, it can be considered that there is a strong relationship between the values of the independent variables and those of the dependent variable, and the determined model can be considered satisfactory in a complex context;

- For the sample made of the protopasta material:

(14) $$l_{\theta }=57267904.87f^{0.351}r^{-0.68}\theta^{-4.925}$$

In the case of the protopasta material, the residual sum of squares had the value *RSS* = 34.32, the normal Gauss sum *S_G_* = 252.75, and the coefficient of determination *R*^2^ = 0.864;

- For the sample made of the prografen material:

(15) $$l_{\theta }=325182106f^{0.069}r^{-0.95}\theta^{-5.389}$$

For the prografen material, the residual sum of squares had the value *RSS* = 46.43, the normal Gauss sum *S_G_* = 440.4, and the coefficient of determination *R*^2^ = 0.806. It is found that the determined value of the coefficient of determination *R*^2^ is between 0.7 and 0.9, which means that there is a strong relationship between the values of the independent and dependent variables and that the model provides a good explanation of the variation of the dependent variable.

In the empirical mathematical models presented above, the exponents for the input factor *f* (which takes into account the shape of the cross-section through the radial fin), the ratio *r,* and temperature *θ* were determined only to allow calculations to be performed and, respectively, to develop graphical representations of the models.

The values of the constants in the power-type functions were determined by minimizing the sum of squared errors, that is, minimizing the sum of squared differences between the observed and predicted values. The assessment of the adequacy of the proposed empirical mathematical model was made by calculating the coefficient of determination *R*^2^, which is a measure of the quality of the fit of the regression model. The coefficient of determination *R*^2^ is a statistical measure that indicates the proportion of the variance in the dependent variable that is predictable from the independent variables in a regression model. Mathematically, *R*^2^ is defined as one minus the ratio of the sum of squared residuals (errors between the observed and predicted values) to the total sum of squares (the total variation in the observed data). It is considered that the closer the *R*^2^ value is to 1, the more it means that the model explains more of the variance.

In principle, the value of Gauss’s criterion is determined as a ratio in which the numerator is a sum of the squares of the differences between the values determined by using the proposed empirical mathematical model and, respectively, the values of the ordinates corresponding to the experimental results for the same values of the abscissas. The denominator of the ratio represents the difference between the number of experimental trials and the number of constants existing in the proposed empirical mathematical model. It is considered that the lower the value of Gauss’s criterion, the more appropriate the mathematical model considered is for the set of experimental results obtained. Following this observation, it can be considered that when several empirical mathematical models are proposed, the most appropriate model will be the one for which the value of Gauss’s criterion is the smallest.

**Figure 11:**
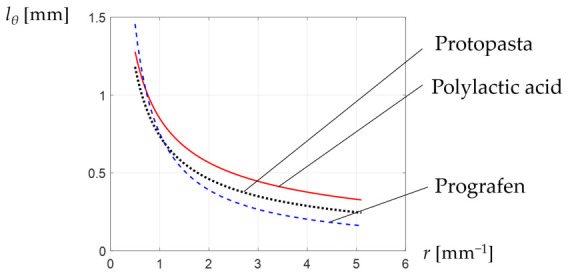
Influence of the ratio *r* of the perimeter and cross-sectional area on the length *l_θ_*, for the three materials (the shape of the component cross-section: circular, which means *f* = 1, *θ* = 40 °C).

**Figure 12:**
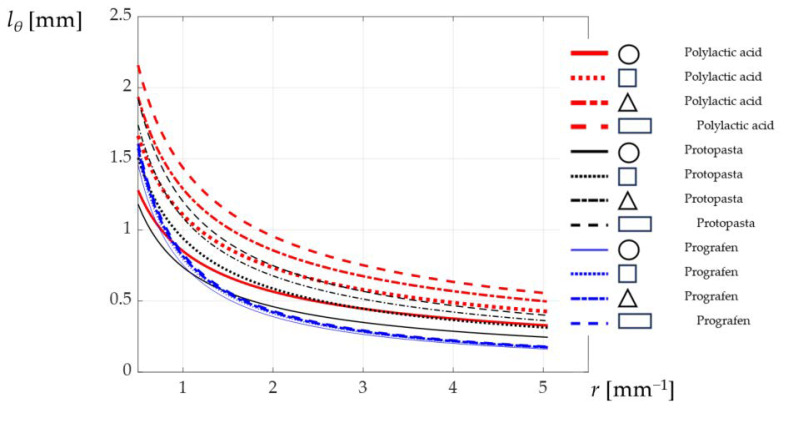
Influence of the ratio r of the perimeter and cross-sectional area of the fin on the length *l_θ_* for the 4 shapes of the cross-section (material: polylactic acid, which means *m* = 1, *f* = 1, *f* = 2, *f* = 3, *f* = 4, *θ* = 40 °C).

**Figure 13:**
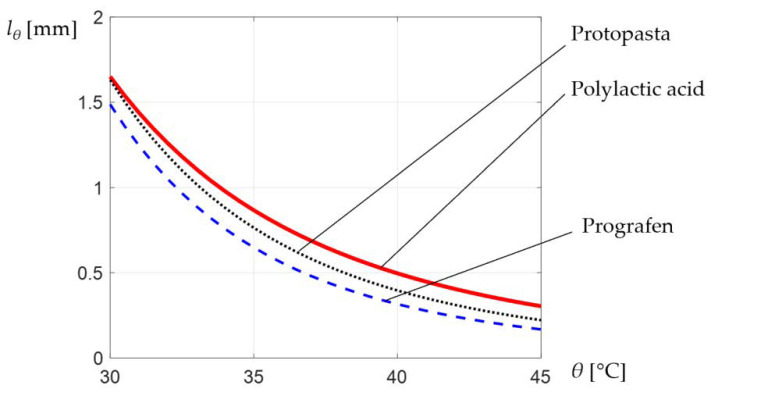
Influence of temperature *θ* on length *l_θ_* for the three materials of the test samples (*m* = 1, *m* = 2, *m* = 3, cross-sectional shape: circular, which means *f* = 1, ratio *r* = 2.5 mm^−1^).

**Figure 14:**
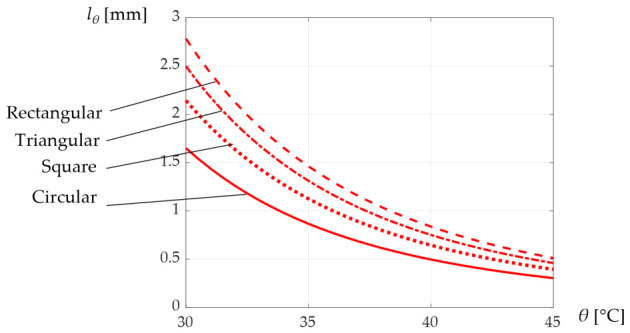
Influence of temperature *θ* on the length *l_θ_*, for the 4 shapes of the cross-section (sample material: polylactic acid, which means *m* = 1, cross-section shape: circular, which means *f* = 1, ratio *r* = 2.5 mm^−1^).

Using the three empirical mathematical models of power function type, the diagrams in Figure 11, Figure 12, Figure 13 and Figure 14 were developed.

## 4. Discussion

In FDM parts, interlayer bonding strength is typically inferior to intralayer strength due to incomplete polymer chain diffusion across deposited filaments. When the principal stress components induced by thermal gradients and mechanical constraints are more closely aligned with the filament deposition direction, as is often the case in vertically printed configurations, the effective load-bearing capacity of the structure increases, resulting in a higher safety factor. Conversely, in the horizontally printed scenario, a greater proportion of the thermo-mechanical stresses may act normal to the layer interfaces, where interlayer adhesion governs failure mechanisms. This leads to slightly elevated equivalent stresses relative to the material strength in the weakest direction, thereby reducing the global safety factor compared to the vertical configuration. The obtained minimum safety factor values of 2.1557 for the PLA FDM Horizontal printing scenario and 2.3256 for the PLA_FDM_Vertical scenario indicate that, in both cases, the component retains a safety margin against failure. From a structural reliability perspective, both configurations can be classified as operating within a conservative stable regime under the investigated thermal conditions. Safety factor values in the range of approximately 2.1–2.3 are not out of the ordinary for polymer-based components subjected to coupled thermal and mechanical loading, particularly when temperature-dependent material properties are incorporated into the numerical model.

For the present study, the authors acknowledge two limitations of the adopted model. The elastic moduli are assumed to be temperature-independent, whereas the thermal conductivity and CTE are treated as temperature-dependent. This internal inconsistency is acknowledged as a simplification and is expected to introduce a modest underestimation of the compliance at temperatures approaching *Tg*, representing a conservative error with respect to deformation prediction. The authors recognize that within this regime, the linear elastic model operates at the boundary of its valid range, where stress redistribution due to localized yielding, which the elastic formulation cannot capture, may modify the peak stress distribution. This limitation is acknowledged as a direction for future refinement, in which an elastoplastic or viscoelastic constitutive description would provide a more physically complete representation of the material response.

By assuming that a single isotropic *k* value biases the predicted temperature gradient, the authors acknowledge simulation minimizes the resistance to heat flow in the build direction, thus overestimating the effective thermal reach of the fin relative to what is actually observed by the IRT camera.

The HTI HT-18 thermal camera, with a manufacturer-stated measurement accuracy of ±2 °C, introduces a non-negligible measurement uncertainty when the temperature differences being resolved along the fin length span a range of approximately 25 °C between ambient and fin base temperature. A ±2 °C uncertainty corresponds to approximately 8% of this full-scale range, which, when applied to temperature threshold measurements such as the distances *l*_40_, *l*_33_, and *l*_28_ used as the primary output parameter of the experimental study, is sufficient to generate positional uncertainties in the measured distances, particularly for the smaller fin geometries where temperature gradients are steepest.

It can be observed that under certain experimental conditions (Figure 13), the fastest thermal transfer was carried out by the polylactic acid samples, followed by those made of protopasta and those made of prografen. It was thus found that, for radial fins with a circular cross-section characterized by the ratio *r* = 0.67 and for a temperature *θ* = 40 °C, the length *l_θ_* corresponding to protopasta is 14% larger than that in the case of protopasta, and that corresponding to polylactic acid is 42% larger than in the case of prografen.

A verification of the validity of the proposed empirical mathematical models is possible, for example, by considering the proper situations. Thus, in the case of a radial fin made of polylactic acid (for which the thermal conductivity is *k* = 0.13 W/m∙K, with a length *L* = 10 mm and having a triangular section, characterized by a side length *a* = 8.06 mm, by a temperature at the base of the fin of 46.3 °C, an ambient temperature of 20 °C, which cools in the air (*h* = 8 W/m^2^∙K), and the tip of the fin is not adiabatic, it is first necessary to determine the cross-sectional area of the radial arm, reaching the value $A=\sqrt{3}a^{2}/4\;=$ 2.81∙10^−5^ m^2^, the perimeter of the triangle being *p* = *3a* = 0.02418 m. Subsequently, using Equation (5), the value of the constant *m* can be determined, reaching a value of *m* ≈ 230 m^−1^. It becomes possible, in this way, to calculate, using Equation (12), the values of temperature *T*(*x*) for different values of distance *x* (this having values between 1.9 and 13.3 mm). By using the values thus obtained, a numerical mathematical model of power function type was first determined, corresponding to the use of Equation (12). A mathematical model of power function type was preferred to be able to possibly compare the values of the exponent attached to the independent variable *θ* in the case of the analytical numerical model with those in the case of the empirical model. It was considered that the values of these exponents provide information regarding the intensity of the influence exerted by the temperature *θ* on the distance *l_θ_* at which the respective temperature *θ* is reached. It was thus established that the mathematical numerical model obtained, of power function type and corresponding to Equation (12), is of the form:

(16) $$l_{\theta }=3.61\cdot 10^{9}\theta^{-5.709}\;$$

for which the value of the coefficient of determination is *R*^2^ = 0.9202. An even higher value of the coefficient of determination is obtained by using a first-degree polynomial mathematical model:

(17) $$l_{\theta }=-0.625\theta +28.385$$

in which case the value of the coefficient of determination is *R*^2^ = 0.9837.

**Figure 15:**
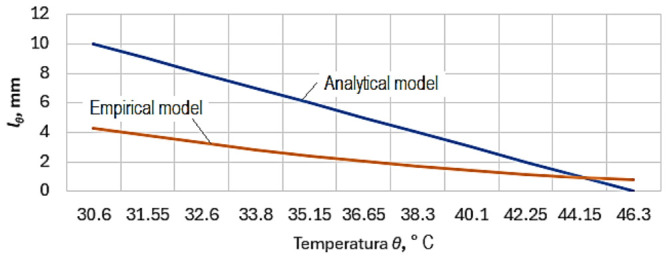
The influence of temperature *θ* on the distance *l_θ_*, according to the analytical Equation (11) and the empirical mathematical model constituted by Equation (13), for a fin having a length *L* = 10 mm and a cross-section in the shape of an equilateral triangle with side *a* = 8.06 mm, assuming an adiabatic tip of the fin, the fin material being polylactic acid.

By taking into account Equations (16) and (17), valid for specified conditions, the graphic representation in Figure 15 was developed. The analysis of this graphic representation shows that for low temperature values (for example, for *θ* = 30 °C), there are differences of about 6 mm between the lengths *l_θ_* calculated using the empirical mathematical model corresponding to Equation (13) and, respectively, using the numerical model corresponding to Equation (17). However, the differences are significantly reduced if high temperature values are taken into account. For example, for a temperature value of 42 °C, the difference between the values obtained using the two models is below 1 mm. A possible explanation for these differences can be formulated by taking into account the anisotropic nature of the polymer material of the fin, with a thermal conductivity that can have different values in distinct directions of heat propagation [59,60,61].

The anisotropy of polymeric materials, especially polymeric composite materials in parts manufactured by 3D printing, is also a factor that introduces differences between theoretical and experimental models related to the development of thermal processes. In the case of polymeric composite materials, the fact that thermal conductivity is not a constant physical quantity, but changes depending on the measurement direction, has led to the consideration of the anisotropy of polymeric composite materials as a tensor quantity [62,63,64,65,66,67,68,69]. The different values of thermal conductivity along distinct directions can be determined by the orientation of the reinforcing materials, the layered structure obtained by 3D printing, and the method of generating layers through a certain 3D printing process. However, for the sake of obtaining simpler empirical mathematical models, the anisotropy of the polymer materials used was not taken into account in the experimental research presented in this paper.

**Figure 16:**
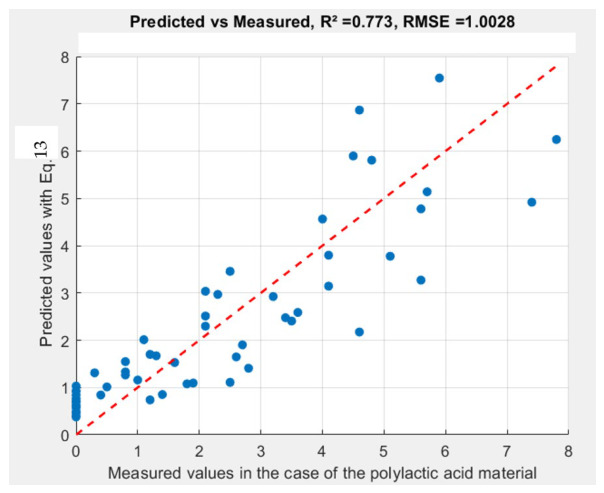
Residual plot for Equation (13) in the case of the polylactic acid material.

**Figure 17:**
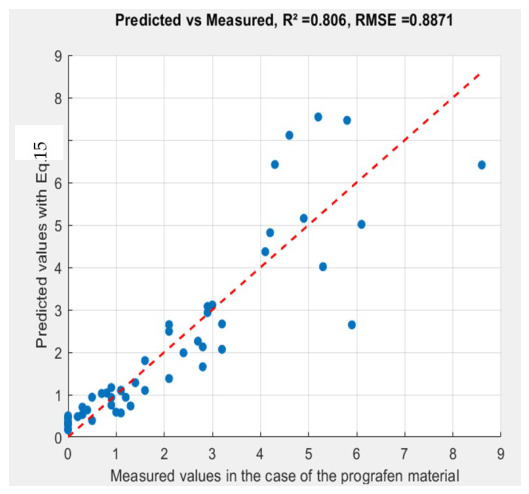
Residual plot for Equation (15) in the case of the prografen material.

The residual plot graphical representation, valid for the empirical mathematical model constituted by Equation (13), can be seen in Figure 16. Another residual plot graphical representation (Figure 17) was developed taking into account the experimental results valid for the case of prografen samples, which led to the empirical mathematical model constituted by Equation (15). These graphical representations provide an image of the dispersion of the experimental results. The analysis of the experimental results listed in Table 3, the empirical mathematical models, and the graphical representations developed on their basis allowed for the formulation of the observations mentioned below.

If the empirical mathematical models are taken into account, it is found that in all three mathematical functions, the exponent with the highest absolute value is the one attached to the input factor temperature *θ*. This means that the strongest influence on the value of the analyzed output parameter *l_θ_* is exerted by the temperature value *θ*. Since the attached exponent has a negative value, for a certain investigated situation, it is found that the highest temperature *θ* value corresponds to the area near the solid body from which the radial fin detaches. This observation confirms the initial hypothesis, according to which the temperature value at the base of the radial component will significantly influence the distance *l_θ_* at which a certain temperature value *θ* is observed, at least for the interval considered for the temperature variation.

The second factor influencing the distance *l_θ_* at which a certain value of temperature *θ* is observed is the ratio *r*, since the absolute value of the exponent attached to the ratio *r* of the cross-section in empirical mathematical models of the power function type is lower than the absolute value of the exponent attached to temperature *θ* as an input factor in the investigated process.

The third input factor, namely the shape of the cross-section through the radial fin (factor *f*), exerts the least influence, since in all three empirical mathematical models, the values of the exponent attached to the input factor *f* have the lowest absolute values.

The exponents attached to the ratio *r* of the cross-sectional area of the radial fin have negative values, which would mean that as the values of these input factors increase, there will be a decrease in the distance *l_θ_* at which a certain value of the temperature *θ* is observed.

In principle, according to the empirical mathematical models determined, as the distance *l_θ_* from the fin base increases, the temperature differences recorded for different materials are lower. For example, according to the information in Figure 13, for a distance *l_θ_* from the fin base of 1.5 mm, the difference between the temperatures of the polylactic acid and prografen samples is about 0.7 °C, while for a distance *l_θ_* of 0.5 mm, this difference is 1.2 °C. As mentioned, in the experimental research, no observations were made regarding the transfer of heat to the external environment by radiation, and this constitutes a limitation of the validity of the results obtained. Another limitation of the validity of the empirical mathematical models determined comes from the fact that these models do not take into account the large variation in some characteristics of the materials incorporated in the fins, which is due to the use of certain conditions for the 3D printing process. For this reason, the results obtained correspond only to those characteristics of the materials generated by using certain values of the 3D printing parameters. Based on the experimental results included in Table 3 and the graphical representations in Figure 11 and Figure 13, it can be concluded that the most intense heat transfer corresponds to the polylactic acid-type polymeric material, for which the highest values of the distance *l_θ_* are found. Lower heat transfer capacities are specific to protopasta and prografen-type polymeric materials, respectively, in this order, for which the values of the parameter *l_θ_* under the same experimental conditions are lower.

For all three materials of the samples, the analysis of the values of the statistical parameters calculated when establishing the empirical mathematical models highlights quite close values, which confirms a fairly similar behavior of the respective materials from the point of view of the general heat transfer process. Thus, it is known that the residual sum of squares *RSS* parameter provides information on the differences between the observed values, i.e., those determined experimentally, and those predicted by the empirical mathematical models (regression models). Since the value of the *RSS* parameter is higher in the case of polylactic acid samples (*RSS* = 58.32), it can be stated that the variability of the experimental results was higher in the case of this material than in the case of prografen and protopasta-type polymeric materials (for which the values of the residual sum of squares were *RSS* = 46.43 and *RSS* = 34.32, respectively).

The Gaussian normal sum *S_G_* has a higher value (*S_G_* = 527.35) in the case of the polylactic acid material, one explanation being that, in the case of this material, a greater dispersion of the experimental results was found than in the case of the other two polymeric materials (*S_G_* = 252.75 for protopasta and *S_G_* = 440.4 for prografen). The close relative values of the *S_G_* parameter confirm, however, that the determination of the experimental results was carried out with approximately the same precision. In the case analyzed here, the calculated values of the coefficient of determination *R*^2^ (*R*^2^ = 0.773 for test samples made of polylactic acid, *R*^2^ = 0.864 for protopasta test samples and *R*^2^ = 0.806 for samples made of prografen) are quite close and show that over 75% of the variation of the considered output parameter (*l_θ_*) is generated by the variation of the input factors for which the experimental results included in Table 3 were obtained (at least in the case of two materials).

**Figure 18:**
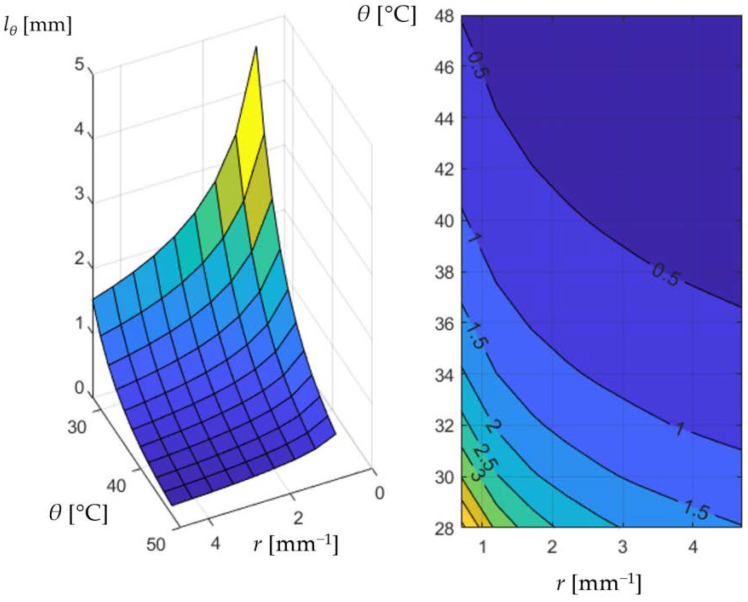
Influence of the size of the ratio *r* of the cross-section through the radial component and the temperature *θ* on the length *l_θ_* at which the temperature *θ* is reached in the case of a polylactic acid sample (*m* = 1), for a circular shape (*f* = 1) of the cross-section through the radial component.

**Figure 19:**
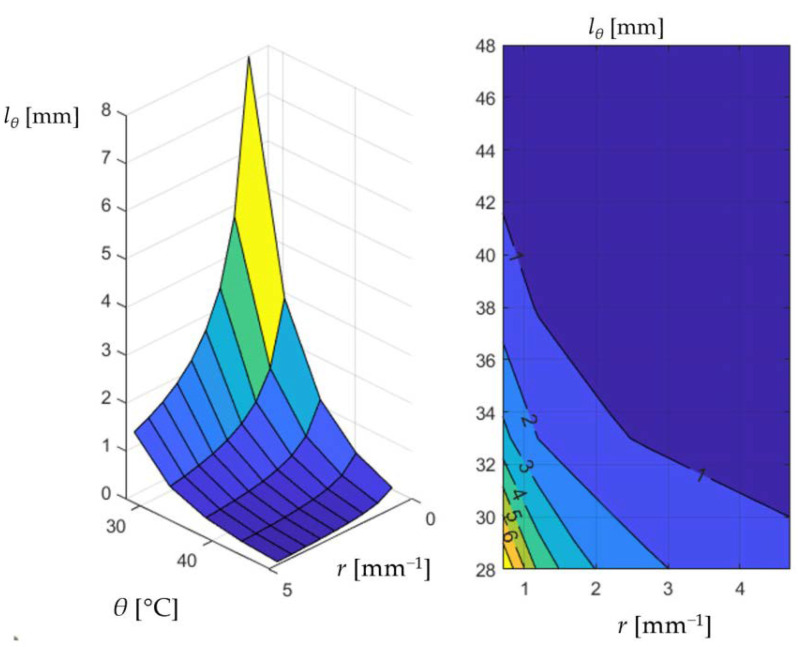
Influence of the size of the ratio *r* of the cross-section through the radial component and the temperature θ on the length at which the temperature θ is reached in the case of a prografen sample (*m* = 3) for a rectangular shape (*f* = 4) of the cross-section through the radial component.

Two spatial coordinate diagrams (Figure 18 and Figure 19) were developed to highlight the variation of the length *l_θ_* as a function of the ratio *r* of the cross-sectional area through the radial component and, respectively, as a function of the temperature *θ* for the prografen material (*m* = 3) and for the polylactic acid (*m* = 1) corresponding to a rectangular (*f* = 1) and circular (*f* = 1) cross-section, respectively.

The experimental results used to identify empirical mathematical models are valid only for the conditions in which the experimental tests took place, but they provide information on how the temperature distribution occurs along the length of a fin made of a certain polymeric material and under predetermined test conditions.

According to the images taken when using the infrared camera in Figure 10 and, in fact, also from the experimental results, it is found that a more intense heat transfer takes place at the base of the fin, where the temperature variation is higher. This observation is also confirmed by other researchers [46].

The temperature distribution in the radial fin is also dependent on the duration of the time interval from the start of the heating process, as well as on the emissivity characteristics of the fin material. Such aspects have not been taken into account, for now, and they constitute limitations of the proposed empirical mathematical models.

On the other hand, a certain limitation of the validity of the observations resulting from the experimental research results from the fact that the variation in the thermal properties of the polymeric materials with the variation in the temperature was not taken into account [70,71,72,73]. It is known that the thermal conductivity, calorific capacity, coefficient of thermal expansion, emissivity, etc., can be strongly affected by the variation in the temperature. In the research undertaken, to avoid overly complex and difficult-to-analyze models, such variations in the temperature were not taken into account, and this fact may influence, to a certain extent, the validity of the results obtained.

It is necessary to mention that in the consulted specialized literature, there is ample information regarding heat transfer through fins made mainly of metallic materials, while references to the behavior of fins made of polymeric materials are relatively few. In principle, the mathematical models developed by other researchers confirm, as expected, the decrease in temperature along fins made of different materials, according to distinct laws of variation of the cross-sectional shape, but corresponding to a variation similar to that established experimentally and presented in the paper for the polymeric materials from which the samples were made [19,20,25,27,29,30,42,70]. Thus, Agarwal et al. proposed the use of a power-type function to describe the variation of the fin surface heat transfer coefficient in the case of air-cooled engine fins, the exponent attached to the fin length having a value of −0.14 [74].

Somewhat contradictory information exists regarding heat transfer through fins made of metallic materials with different cross-sectional shapes. For example, the proposed mathematical models allow the formulation of a conclusion that a heat transfer takes place with a minimum consumption of material in the case of triangular sections of the fins [69,70], while in other cases, the existence of a more intense heat transfer has been highlighted in the case of fins with rectangular sections [69]. In other situations, it is stated that elliptical fins can ensure a higher efficiency of heat transfer compared to, for example, triangular and rectangular fins [72]. In accordance with the results presented in Figure 14, it can be observed that the fin length at which a temperature *θ_b_* is reached has the highest values in the case of polylactic acid fins with a rectangular cross-section.

In relation to the information available in the specialized literature accessed, the results presented in this paper provide an image of the simultaneous temperature variation along the radial fins of the central part, as a result of heat dissipation from the heated central bushing to the free ends of the radial fins.

## 5. Conclusions

The objective of the research was to obtain additional information on the simultaneous propagation of heat through radial fins with cross-sections of different shapes and sizes. Test samples made of three distinct polymer materials (polylactic acid (PLA)-based materials, i.e., standard PLA, PLA with carbon black, and PLA with graphene) and having radial fins with four cross-sectional shapes (circular, square, triangular, and rectangular) were manufactured by 3D printing. The use of an infrared camera allowed for obtaining images of how heat propagates through the radial fins from the central area. By mathematically processing the experimental results, empirical mathematical models were determined. These models highlight the influence of the shape and cross-sectional area of the radial fins and, respectively, the temperature reached at a certain length of the radial fin measured from the central area. The research results could be used in the design of parts made of polymeric materials that would have radial fins and would be subjected to heating processes from the area of joining the fins to the central part. Such fins can be found in electronics, fine mechanics, the medical field, etc. The main observations resulting from the analysis of the empirical mathematical models are the following:

1. It can be seen that the results obtained confirmed some of the initial hypotheses. Compared to the results of previous studies, in this case, the simultaneous heating of fins with different shapes and sizes of cross-sections through the fins was taken into account, from the central bushing to which the fins were attached.
2. For the experimental conditions taken into account, it was found that thermal transfer takes place most rapidly in the fin with a rectangular cross-section, followed by the triangular, square, and circular shapes. Thus, under certain experimental conditions (40 °C), in the case of the polylactic acid sample, the heat transfer characterized by the length *l_θ_* in the case of a rectangular section is 6% larger than in the case of a triangular section, 23% larger than in the case of a square section, and 60% larger than in the case of a circular section.
3. At the same time, among the three polymer materials from which the samples were manufactured by 3D printing, it was found that the fastest thermal transfer was carried out by the polylactic acid samples, followed by those made of protopasta and those made of prografen. Thus, it was found that, for radial component with a circular cross-section having the ratio *r* = 0.67, the length *l_θ_* corresponding to protopasta is 14% larger than that in the case of prografen, and that corresponding to polylactic acid is 42% larger than in the case of prografen.
4. The safety factor values obtained are commonly regarded as appropriate for thermoplastics like PLA, which exhibit pronounced softening, stiffness degradation, and thermal expansion as the temperature approaches the glass transition region. FEM revealed that in both cases, the component retains a safety margin against failure with approximately 46–48% of the effective allowable stress for the horizontally printed part and approximately 43% for the vertically printed part.
5. The main limitations that may affect the validity of the results come from the failure to take into account, to a great extent, some factors that may exert influence on thermal transfer through the fins made of polymeric materials manufactured by 3D printing. Thus, the empirical mathematical models determined do not take into account the air circulation around the fins nor the characteristics of the materials incorporated in the fins, influenced by the conditions of the 3D printing process.
6. The absence of experimentally measured *k*(*T*) data for the specific grades of material used, as well as the qualitative nature of the FEM-to-IRT comparison, is seen as a limitation whose removal would require experimentally measured *k*(*T*) curves obtained by methods such as laser flash analysis or the transient plane source technique, which are not available at this time. This limitation constrains the quantitative accuracy of the absolute temperature values predicted by the FEM model and reinforces the necessity of presenting the FEM results as qualitative indicators of the spatial pattern of temperature distribution, rather than as quantitatively accurate temperature field predictions.
7. In the future, it is intended to expand the categories of polymer materials used to make the test samples and possibly determine other empirical mathematical models that could better characterize heat transfer through the respective polymer materials. Other research directions that could be addressed in the future could be the validation of experimental results using more adequate numerical simulations and, respectively, the investigation of heat transfer under transient loading conditions.
8. For the three materials studied in the present work, thermal anisotropy is expected to be particularly pronounced for the prografen material, since graphene flakes are known to orient preferentially along the raster direction during extrusion, producing a large contrast between the in-plane (high) and through-thickness (low) components of the thermal conductivity tensor.

## Figures and Tables

**Table 1:** Results for mesh sensitivity check.

**Horizontal Configuration**						
**Element size**	**Nodes**	**Elements**	**Max stress (MPa)**	**Δ Stress %**	**Max def. (mm)**	**Δ Def. %**
5.0 mm	279,427	190,382	24.3370	-	0.0091	-
4.0 mm	281,313	191,882	22.8686	6.03%	0.0091	0.00%
3.0 mm	283,588	191,893	21.7459	4.91%	0.0091	0.00%
2.5 mm	286,548	196,382	20.9492	3.66%	0.0090	1.10%
2.0 mm	294,781	201,363	19.9479	4.78%	0.0090	0.00%
1.0 mm	367,703	250,187	16.6998	16.28%	0.0089	1.11%
**Vertical configuration**						
**Element size**	**Nodes**	**Elements**	**Max stress (MPa)**	**Δ Stress %**	**Max def. (mm)**	**Δ Def. %**
5.0 mm	279,431	190,860	23.3964	-	0.0091	-
4.0 mm	283,826	198,741	22.1321	5.40%	0.0091	0.00%
3.0 mm	290,186	202,919	20.9172	5.49%	0.0091	0.00%
2.5 mm	292,248	219,451	20.0403	4.19%	0.0090	1.10%
2.0 mm	298,582	201,363	19.2706	3.84%	0.0090	0.00%
1.0 mm	367,542	250,035	15.2776	20.72%	0.0089	1.11%

**Table 2:** Characteristics of materials used for 3D printing of test samples.

Material	Characteristics of the Materials Used to Make the Samples						
	Tensile Strength	Maximum Elongation	Young’s Modulus	Composition	Features of Use	Thermal Conductivity, W∙m^−1^∙K^−1^	Density, kg/m^3^
Polylactic acid (PLA)	50 MPa	6%	3500 MPa	PLA (pure)	Easy to use, good adhesion	0.06426–0.09044 [36]	1.24
Protopasta	14.2 MPa [10]	2.4% [10]	1000 MPa [10]	PLA + carbon black	Electrically conductive	0.25 [37]	1.15
Prografen	58.4 MPa	4.4%	1754 MPa	PLA + graphene flake	Rigid, low thermal shrinkage	1–10 [38]	1.24

**Table 3:** Conditions for carrying out experimental tests and results obtained.

Exp. no.	Material, *m*	Cross-Section Shape, *f*	Characteristic Dimensions, *d*, *l* × *l*, *l* × *l* × *l*, *l* × *h* (mm)	Ratio *r* Between the Perimeter *p* (mm) and the Area *A* (mm^2^), mm^−1^	Cross-Sectional Area of the Test Sample, *S*, mm^2^	The Distance *l_θ_* at Which a Certain Temperature *θ* is Sensed (mm)			
						46.3 °C	40 °C	33 °C	28 °C
Row no. 1	2	3	4	5	6	7	8	9	10
1	PLA (*m* = 1)	Circular (*f* = 1)	Ø 1	4.00	0.785	-	0	0.4	1.3
2	PLA (*m* = 1)	Circular (*f* = 1)	Ø 2	2.00	3.142	-	0	0.8	2.1
3	PLA (*m* = 1)	Circular (*f* = 1)	Ø 4	1.00	12.566	0	1.4	2.7	5.1
4	PLA (*m* = 1)	Circular (*f* = 1)	Ø 6	0.67	28.274	0	1.8	3.5	5.6
5	PLA (*m* = 1)	Square (*f* = 2)	0.88 × 0.88	4.55	0.774	-	0	0.5	1.1
6	PLA (*m* = 1)	Square (*f* = 2)	1.77 × 1.77	2.26	3.133	-	0	1.6	2.1
7	PLA (*m* = 1)	Square (*f* = 2)	3.55 × 3.55	1.13	12.53	-	0	2.1	4
8	PLA (*m* = 1)	Square (*f* = 2)	5.31 × 5.31	0.75	28.2	0	0.3	3.2	4.8
9	PLA (*m* = 1)	Equilateral triangle (*f* = 3)	1.34 × 1.34 × 1.34	5.17	0.77	-	0	1.9	4.6
10	PLA (*m* = 1)	Equilateral triangle (*f* = 3)	2.69 × 2.69 × 2.69	2.6	3.08	0	1.2	2.6	5.6
11	PLA (*m* = 1)	Equilateral triangle (*f* = 3)	5.38 × 5.38 × 5.38	1.29	12.53	0	2.5	3.4	7.4
12	PLA (*m* = 1)	Equilateral triangle (*f* = 3)	8.06 × 8.06 × 8.06	0.86	28.12	0	2.8	4.1	7.8
13	PLA (*m* = 1)	Rectangular (*f* = 4)	9.42 × 3	0.88	28.26	0	0.8	2.5	4.6
14	PLA (*m* = 1)	Rectangular (*f* = 4)	14.13 × 2	1.14	28.26	0	0.8	2.3	4.5
15	PLA (*m* = 1)	Rectangular (*f* = 4)	18.84 × 1.5	1.44	28.26	0	1	3.6	5.7
16	PLA (*m* = 1)	Rectangular (*f* = 4)	5.65 × 5	0.75	28.25	0	1.2	4.1	5.9
17	Protopasta (*m* = 2)	Circular (*f* = 1)	Ø 1	4.00	0.785	-	0	0.3	1.5
18	Protopasta (*m* = 2)	Circular (*f* = 1)	Ø 2	2.00	3.142	-	0	0.7	2.4
19	Protopasta (*m* = 2)	Circular (*f* = 1)	Ø 4	1.00	12.566	0	0.9	2.6	5.9
20	Protopasta (*m* = 2)	Circular (*f* = 1)	Ø 6	0.67	28.274	0	1.2	3.8	6.7
21	Protopasta (*m* = 2)	Square (*f* = 2)	0.88 × 0.88	4.55	0.774	-	0	0.5	1.8
22	Protopasta (*m* = 2)	Square (*f* = 2)	1.77 × 1.77	2.26	3.133	-	0	1.1	2.8
23	Protopasta (*m* = 2)	Square (*f* = 2)	3.55 × 3.55	1.13	12.53	-	0	1.9	5.3
24	Protopasta (*m* = 2)	Square (*f* = 2)	5.31 × 5.31	0.75	28.2	0	0.9	3.3	6.1
25	Protopasta (*m* = 2)	Equilateral triangle (*f* = 3)	1.34 × 1.34 × 1.34	5.17	0.77	-	0	1.8	3.2
26	Protopasta (*m* = 2)	Equilateral triangle (*f* = 3)	2.69 × 2.69 × 2.69	2.6	3.08	0	0.6	2.2	4.3
27	Protopasta (*m* = 2)	Equilateral triangle (*f* = 3)	5.38 × 5.38 × 5.38	1.29	12.53	0	1.4	3.1	5.9
28	Protopasta (*m* = 2)	Equilateral triangle (*f* = 3)	8.06 × 8.06 × 8.06	0.86	28.12	0	1.5	4	6.1
29	Protopasta (*m* = 2)	Rectangular (*f* = 4)	9.42 × 3	0.88	28.26	0	0.8	2.8	5.2
30	Protopasta (*m* = 2)	Rectangular (*f* = 4)	14.13 × 2	1.14	28.26	0	0.9	3	5.4
31	Protopasta (*m* = 2)	Rectangular (*f* = 4)	18.84 × 1.5	1.44	28.26	0	1.2	3.3	5.9
32	Protopasta (*m* = 2)	Rectangular (*f* = 4)	5.65 × 5	0.75	28.25	0	0.9	3.2	5.8
33	Prografen (*m = 3*)	Circular (*f* = 1)	Ø 1	4.00	0.785	-	0	1.1	2.1
34	Prografen (*m* = 3)	Circular (*f* = 1)	Ø 2	2.00	3.142	0	0.5	1.6	3.2
35	Prografen (*m* = 3)	Circular (*f* = 1)	Ø 4	1.00	12.566	0	0.9	2.8	4.9
36	Prografen (*m* = 3)	Circular (*f* = 1)	Ø 6	0.67	28.274	0	1.1	3	5.2
37	Prografen (*m* = 3)	Square (*f* = 2)	0.88 × 0.88	4.55	0.774	-	0	0.3	1.4
38	Prografen (*m* = 3)	Square (*f* = 2)	1.77 × 1.77	2.26	3.133	-	0	0.7	2.1
39	Prografen (*m* = 3)	Square (*f* = 2)	3.55 × 3.55	1.13	12.53	0	0.3	2.4	4.2
40	Prografen (*m* = 3)	Square (*f* = 2)	5.31 × 5.31	0.75	28.2	0	0.8	2.9	4.6
41	Prografen (*m* = 3)	Equilateral triangle (*f* = 3)	1.34 × 1.34 × 1.34	5.17	0.77	-	0	0.2	0.9
42	Prografen (*m* = 3)	Equilateral triangle (*f* = 3)	2.69 × 2.69 × 2.69	2.6	3.08	-	0	0.9	2.7
43	Prografen (*m* = 3)	Equilateral triangle (*f* = 3)	5.38 × 5.38 × 5.38	1.29	12.53	0	0.4	1.6	4.1
44	Prografen (*m* = 3)	Equilateral triangle (*f* = 3)	8.06 × 8.06 × 8.06	0.86	28.12	0	0.5	2.1	4.3
45	Prografen (*m* = 3)	Rectangular (*f* = 4)	9.42 × 3	0.88	28.26	0	1.2	5.9	8.6
46	Prografen (*m* = 3)	Rectangular (*f* = 4)	14.13 × 2	1.14	28.26	0	1.3	3.2	6.1
47	Prografen (*m* = 3)	Rectangular (*f* = 4)	18.84 × 1.5	1.44	28.26	0	1	2.8	5.3
48	Prografen (*m* = 3)	Rectangular (*f* = 4)	5.65 × 5	0.75	28.25	0	1.1	2.9	5.8

## Data Availability

Information supporting the reported results can be obtained upon request from the authors.

## Abbreviations

The following abbreviations are used in this manuscript:

*θ*	Temperature value, in °C
*l_θ_*	Distance at which a certain temperature *θ* is reached, in mm
*m*	Nature of the polymer material
*f*	Shape of the cross-section

## References

[1] Fischer A.J., Zhong Y., Zhang L., Wu W., Drummer D. (2019). Heat propagation in thermally conductive polymers of PA6 and hexagonal boron nitride. Fire Mater..

[2] Cai Z., Thirunavukkarasu N., Diao X., Wang H., Wu L., Zhang C., Wang J. (2022). Progress of Polymer-Based Thermally Conductive Materials by Fused Filament Fabrication: A Comprehensive Review. Polymers.

[3] Czel G., Sycheva A., Janovszky D. (2023). Effect of different fillers on thermal conductivity, tribological properties of Polyamide 6. Sci. Rep..

[4] Shanmugam V., Babu K., Kannan G., Mensah R.A., Samantaray S.K., Das O. (2024). The thermal properties of FDM printed polymeric materials: A review. Polym. Degrad. Stab..

[5] Hrițuc A., Mihalache A.M., Slătineanu L., Dodun O., Nagîț G. (2023). Heat Transfer in 3D-Printed Polymer Cylindrical Parts. Macromol. Sympos..

[6] Hriţuc A., Dodun O., Duşa P., Mihalache A., Nagîţ G., Slătineanu L. (2023). Identifying a device for tracking the evolution of thermal transfer in 3d printed parts using principles from axiomatic design. Proceedings of the 15th International Conference on Axiomatic Design 2023.

[7] Reynolds B.W., Fee C.J., Morison K.R., Holland D.J. (2022). Characterisation of heat transfer within 3D printed TPMS heat exchangers. ISSRN Electron. J..

[8] Ly D.V., Kishi Y., Nakayama T., Yamada N. (2024). Thermal performance of polymer gyroid heat exchangers combined with phase change materials as a latent heat thermal energy storage system: An experimental investigation. Int. J. Heat Mass Transf..

[9] Lalot S., Tournier C., Jensen M. (1999). Fin efficiency of annular fins made of two materials. Int. J. Heat Mass Trans..

[10] Heinle C., Drummer D. (2010). Potential of thermally conductive polymers for the cooling of mechatronic parts. Phys. Procedia.

[11] Marchetto D.B., Moreira D.C., Ribatski G. (2018). A review on polymer heat sinks for electronics cooling applications. Proceedings of the 17th Brazilian Congress of Thermal Sciences and Engineering, Águas de Lindóia, Brazil, 25–28 November 2018.

[12] Yu S.-H., Lee K.-S., Yook S.-J. (2010). Natural convection around a radial heat sink. Int. J. Heat Mass Trans..

[13] Kanthimathi T., Naga S.R.G. (2016). Heat transfer through annular composite fins. J. Mech. Eng. Technol..

[14] Lee J.B., Kim H.J., Kim D.-K. (2016). Experimental study of natural convection cooling of vertical cylinders with inclined plate fins. Energies.

[15] Mathiazhagan P., Jayabharathy S. (2012). Heat transfer and temperature distribution of different fin geometry using numerical method. JP J. Heat Mass Transf..

[16] Asadi M., Khoshkho R.H. (2013). Temperature distribution along a constant cross sectional area fin. Int. J. Mech. Appl..

[17] Khaled R.A. (2013). Generalized correlations for heat transfer through high performance fins. Adv. Mech. Eng..

[18] Madhura B.K.R., Rajath G.K., Singh P., Gupta R.K., Ray K., Bandyopadhyay A. (2021). Investigation on thermal distribution and heat transfer rate of fins with various geometries. Proceedings of International Conference on Trends in Computational and Cognitive Engineering.

[19] Karwa R. (2020). Extended surfaces (fins). Heat and Mass Transfer.

[20] Khan N.A., Sulaiman M., Alshammari F.S. (2022). Heat transfer analysis of an inclined longitudinal porous fin of trapezoidal, rectangular and dovetail profiles using Cascade neural networks. Struct. Multidiscip. Optim..

[21] Haq K., Shah K., Abdeljawad T. (2023). Analysis of periodic heat transfer through extended surfaces. Therm. Sci..

[22] Cui Q., Huang X., Wang X., Wu C., Su J. (2024). Evaluation of a novel annular fin for heat transfer enhancement in hot water oil-displacement system. Therm. Sci. Eng. Prog..

[23] Habib S., Waseem, Khan Z., Boulaaras S., Rahman M.U., Islam S., Guefaifia R. (2024). Analysis of the thermal distribution of a porous radial fin influenced by an inclined magnetic field with neural computing. Sci. Rep..

[24] Keshtiban M.M., Targhi M.Z., Heyhat M.M. (2024). Effects of radial fin arrangements on the thermal performance of turbulent liquid jet impingement heat sinks: Experimental and numerical approach. Appl. Therm. Eng. (Part C).

[25] Kumar R.S.V., Sowmya G., Abhilasha S.K., Prasannakumara B.C. (2025). Solution by operational matrix based Vieta-Lucas collocation method to analyze the thermal performance of the convex spine fin applicable in air conditioning systems. Int. Commun. Heat Mass Trans..

[26] Bagatella S., Cereti A., Manarini F., Cavallaro M., Suriano R., Levi M. (2024). thermally conductive and electrically insulating polymer-based composites heat sinks fabricated by fusion deposition modeling. Polymers.

[27] Manvitha N.V., Gireesha B.J., Gowtham K.J. (2025). Taylor wavelet solution procedure for the analysis of triangular porous fin made of functionally graded materials in the presence of hybrid nanofluid. Acta Mech..

[28] Souza R., Nobrega G., Afonso I.S., Pereira J., Cardoso E., Marques F., Vilarinho C., Moita A., Lima R.A. (2025). Thermal performance evaluation of pure PDMS and PDMS composites heat exchangers. J. Therm. Anal. Calorim..

[29] Li L., Tang Q., Chen X., Weng C. (2025). Polymer-based pin-fin microchannel heat exchangers: A comparative study of material and structural effects on performance. Int. J. Therm. Sci..

[30] Manvitha N.V., Gireesha B.J., Gowtham K.J. (2026). Efficiency scrutinization of fully wet porous inclined conical spine with varying surface emissivity: A Fibonacci wavelet collocation approach. Int. J. Mech. Mater. Des..

[31] Cengel Y.A. (1997). Heat Transfer. A Practical Approach.

[32] Incropera F.P., DeWitt D.P., Bergman T.L., Lavine A.S. (2007). Fundamentals of Heat and Mass Transfer.

[33] Holman J.P. (2009). Heat Transfer.

[34] Hahn D.W., Özisik M.N. (2012). Heat Conduction.

[35] Ma J., Sun Y., Li S. (2022). Element differential method for non-Fourier heat conduction in the convective-radiative fin with mixed boundary conditions. Coatings.

[36] Barkhad M.S., Abu-Jdayil B., Mourad A.H.I., Iqbal M.Z. (2020). Thermal insulation and mechanical properties of polylactic acid (PLA) at different processing conditions. Polymers.

[37] Blanco I., Cicala G., Recca G., Tosto C. (2022). Specific heat capacity and thermal conductivity measurements of PLA-based 3D-printed parts with milled carbon fiber reinforcement. Entropy.

[38] Balandin A.A. (2011). Thermal properties of graphene and nanostructured carbon materials. Nat. Mater..

[39] Ozisik M.N. (1985). Heat Transfer: A Basic Approach.

[40] Muhammad A.K., Mohammed T.W., Resan K.K. (2025). Thermal conductivity of porous plastics manufactured by 3D printing: Controlling the design of the cavities and corresponding effects. J. Ther. Eng..

[41] Tychanicz-Kwiecien M., Grosicki S., Markowicz M. (2025). Experimental investigation of thermal conductivity of selected 3D-printed materials. Materials.

[42] Kader A.H.A., Latif M.S.A., Nour H.M. (2016). General exact solution of the fin problem with variable thermal conductivity. Propuls. Power Res..

[43] Kumar R.S.V., Kumar R.N., Sowmya G., Prasannakumara B.C., Sarris I.E. (2022). Exploration of temperature distribution through a longitudinal rectangular fin with linear and exponential temperature-dependent thermal conductivity using DTM-Pade approximant. Symmetry.

[44] Vijaybabu T.R., Ramesh T., Pandipati S., Mishra S., Sridevi G., Raja C.P., Mensah R.A., Das O., Misra M., Mohanty A. (2023). High thermal conductivity polymer composites fabrication through conventional and 3D printing processes: State-of-the-art and future trends. Macromol. Mater. Eng..

[45] Hassan M.S., Tahseen T.A., Weis M.M. (2022). Natural convection from a radial heat sink with triangular fins. NTU J. Eng. Technol..

[46] Bunjaku F., Buza K., Filkoski R.V. (2025). Comparative analysis of thermal performance and geometric characteristics of tubes with rectangular and triangular fins. Processes.

[47] Ahmed H.E., Salman B.H., Kherbeet A.S., Ahmed M.I. (2018). Optimization of thermal design of heat sinks: A review. Int. J. Heat Mass Trans..

[48] Rahman M.A., Hasnain S.M.M., Paramasivam P., Ayanie A.G. (2024). Advancing thermal management in electronics: A review of innovative heat sink designs and optimization techniques. RSC Adv..

[49] Yan Z., Oon C.S., Tan B.T., Ooi J.B. (2025). Geometric design and optimization of extended surfaces for enhanced heat transfer: A comprehensive review. Results Eng..

[50] Abbas E.F., Jassim A.H., Thamer K.S. (2023). Review of the fin optimization in the heat sink design. Int. Rev. Mech. Eng..

[51] Leontiou T., Fyrillas M.M. (2015). Critical thickness of an optimum extended surface characterized by uniform heat transfer coefficient. arXiv.

[52] Blommaert M., Vangeffelen A., Basaran M., Buckinx G., Baelmans M. (2025). A unit-cell shape optimization approach for maximizing heat transfer in periodic fin arrays at constant solid temperature. Struct. Multidiscip. Optim..

[53] Sobamowo M.G. (2016). Thermal analysis of longitudinal fin with temperature-dependent properties and internal heat generation using Galerkin’s method of weighted residual. Appl. Therm. Eng..

[54] Alfian D.G.C., Silitonga D.J. (2025). Analyzing temperature distribution in multiple fin geometries to optimize heat transfer efficiency. VANOS J. Mech. Eng. Educ..

[55] Karthick A. (2021). Temperature distribution analysis of composite heat sink (pin fin) by experimental and finite element method. J. Manuf. Eng..

[56] Hemanth J. (2017). Experimental, mathematical and finite element analysis (FEA) of temperature distribution through rectangular fin with circular perforations. Model. Simul. Mater. Sci. Eng..

[57] Prajapati H., Ravoori D., Woods R.L., Jain A. (2018). Measurement of anisotropic thermal conductivity and inter-layer thermal contact resistance in polymer fused deposition modeling (FDM). Addit. Manuf..

[58] Ravoori D., Alba L., Prajapati H., Jain A. (2018). Investigation of process-structure-property relationships in polymer extrusion based additive manufacturing through in situ high speed imaging and thermal conductivity measurements. Addit. Manuf..

[59] Pan X., Debije M.G., Albert P.H.J. (2021). Schenning High thermal conductivity in anisotropic aligned polymeric materials. ACS Appl. Polym. Mater..

[60] Kurabayashi K. (2001). Anisotropic Thermal properties of solid polymers. Int. J. Thermophys..

[61] Kim W.-J., An M.-R., Park S.-H. (2025). Reduced anisotropic in thermal conductivity of polymer compositesvia chemically bonded bn–SiC hybrid fillers. Polymers.

[62] Wei X., Wang Z., Tian Z., Luo T. (2021). Thermal transport in polymers: A review. ASME J. Heat Transf..

[63] Han Y., Peng X. (2025). Thermal performance of a moving fin with temperature-dependent thermal conductivity in convective and radiative environment. Heliyon.

[64] Ghasemi S.E., Hatami M., Ganji D.D. (2014). Thermal analysis of convective fin with temperature-dependent thermal conductivity and heat generation. Case Stud. Therm. Eng..

[65] Pawlak S., Tokarski M., Ryfa A. (2022). Measurement of the anisotropic thermal conductivity of carbon-fiber/epoxy composites based on laser-induced temperature field: Experimental investigation and numerical analysis. Int. Commun. Heat Mass Transf..

[66] Yang T., Leng J., Hu J., Wang P., Edeleva M., Cardon L., Yan Z., Wang T., Zhan J. (2023). Enhancing thermal conductivity and balancing mechanical properties of 3D-printed iPP/HDPE-based dielectric composites via the introduction of hybrid fillers and tailored crystalline structure. Virtual Phys. Prototyp..

[67] Mao L.-K., Liu Q., Chen H., Cheng W.-L. (2024). A novel model of the anisotropic thermal conductivity of composite phase change materials under compression. Int. Commun. Heat Mass Transf..

[68] Tan J., Zhang Y. (2024). Thermal conductive polymer composites: Recent progress and applications. Molecules.

[69] Ayoobi A., Ramezanizadeh M., Alhuyi-Nazari M. (2021). Optimization of temperature distribution and heat flux functions for cylindrical and conical micro-fins by applying genetic algorithm. J. Therm. Anal. Calorim..

[70] dos Santos W.N., de Sousa J.A., Gregorio R. (2013). Thermal conductivity behaviour of polymers around glass transition and crystalline melting temperatures. Polym. Test..

[71] Asfew K.N., Ivens J., Moens D. (2022). Temperature dependence of thermophysical properties of carbon/polyamide410 composite. Funct. Compos. Mater..

[72] Shardakov I.N., Trufanov A.N. (2021). Identification of the Temperature Dependence of the Thermal Expansion Coefficient of Polymers. Polymers.

[73] Breitkopf C. (2024). Theoretical characterization of thermal conductivities for polymers—A review. Thermo.

[74] Agarwal P., Shrikhande M., Srinivasan P. Heat transfer simulation by CFD from fins of an air cooled motorcycle engine under varying climatic conditions. Proceedings of the World Congress on Engineering 2011.

